# Complexation Preferences of Dynamic Constitutional Frameworks as Adaptive Gene Vectors

**DOI:** 10.1002/chem.202203062

**Published:** 2022-12-19

**Authors:** Dan‐Dan Su, Virginie Gervais, Sébastien Ulrich, Mihail Barboiu

**Affiliations:** ^1^ Institut Européen des Membranes Adaptive Supramolecular Nanosystems Group University of Montpellier ENSCM-CNRS Place E. Bataillon CC047 Montpellier 34095 France; ^2^ Institut des Biomolécules Max Mousseron (IBMM) Université de Montpellier CNRS, ENSCM 34095 Montpellier France; ^3^ Université Paris-Saclay CEA, CNRS Institute for Integrative Biology of the Cell (I2BC) 91198 Gif-sur-Yvette France

**Keywords:** biomolecular recognition, dynamic constitutional frameworks, DNA complexation, self-assembly

## Abstract

The growing applications of therapeutic nucleic acids requires the concomitant development of vectors that are optimized to complex one type of nucleic acid, forming nanoparticles suitable for further trafficking and delivery. While fine‐tuning a vector by molecular engineering to obtain a particular nanoscale organization at the nanoparticle level can be a challenging endeavor, we turned the situation around and instead screened the complexation preferences of dynamic constitutional frameworks toward different types of DNAs. Dynamic constitutional frameworks (DCF) are recently‐identified vectors by our group that can be prepared in a versatile manner through dynamic covalent chemistry. Herein, we designed and synthesized 40 new DCFs that vary in hydrophilic/hydrophobic balance, number of cationic headgroups. The results of DNA complexation obtained through gel electrophoresis and fluorescent displacement assays reveal binding preferences of different DCFs toward different DNAs. The formation of compact spherical architectures with an optimal diameter of 100–200 nm suggests that condensation into nanoparticles is more effective for longer PEG chains and PEI groups that induce a better binding performance in the presence of DNA targets.

## Graphical abstract

**Dynamic constitutional frameworks**: 40 different amphiphilic dynamic constitutional frameworks were prepared in a stepwise covalent self‐assembly process and their DNA complexation preferences revealed marked differences, not only as a function of the number and type of cationic headgroups, but also as a function of the type of DNA, yielding distinct polyplex architectures.

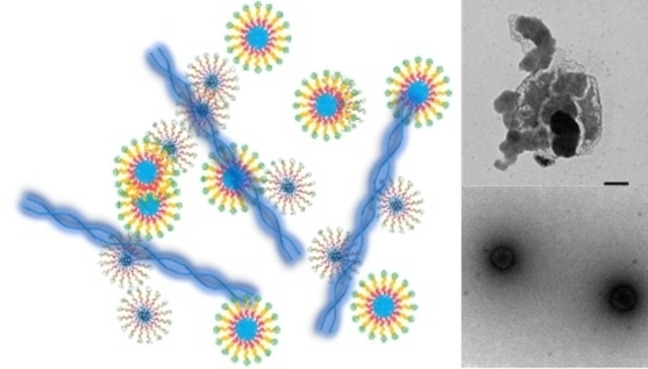

## Introduction

The effective and safe delivery of therapeutic nucleic acids has become an important objective in an ever‐increasing number of applications. Indeed, the diversity of nucleic acids having therapeutic potential has grown significantly^[1]^ and encompasses long circular DNA (pDNA), short double‐stranded RNA (siRNA), CRISPR‐Cas9 editing tools and mRNA for diverse application in cancer treatment, rare diseases, and Covid‐19 vaccines.^[2]^ Given that nucleic acids are vulnerable to endogenous nuclease enzymes and do not effectively translocate the cell membranes because of their hydrophilic nature, it is of paramount importance to develop vectors that protect them from enzymatic degradation and promote their internalization in cells of interest.

Generally, nucleic acids, as polyanions, can be spontaneously complexed by positively‐charged macromolecules via multivalent electrostatic interactions.^[3]^ However, since the nature of cationic groups, their number, position and density dictate the strength of the interaction, the architecture of these cationic macromolecules is critical, and navigating this vast chemical space can sometimes be like finding a needle in a haystack. Therefore, over the past decades, a multitude of cationic vectors including polyethyleneimine,^[4]^ guanidinium‐based polymers/ dendrimers,^[5]^ dynamic covalent frameworks[^6^, ^7^, ^8^] or dynamic polymers,[^9^, ^10^] and peptides,^[11]^ have been identified as effective nucleic acids complexation agents.^[12]^ Additionally, the idea of using multivalent supramolecular polymers is also gradually gaining attention for achieving cellular uptake.^[13]^ Yet, general structure‐activity relationships remain elusive, due to the dynamic complexity of chemical spaces of both vectors and nucleic acids. Specific knowledge can be developed in a single series of synthetic vectors that can be fine‐tuned by molecular engineering. For instance, cyclodextrin‐based systems for nucleic acids binding and delivery,^[14]^ have been investigated in details structure‐activity relationships.^[15]^ They found that the number of positive groups,^[16]^ hydrophilic/hydrophobic balance,^[17]^ as well as their propensity to form higher‐order self‐assemblies^[18]^ are key for pDNA binding and dictate the nanoscale organization of the resulting polyplexes,^[19]^ hence affect the transfection efficiency both in living cells and in vivo.^[20]^ Thus, differences in the architecture of polyplexes have a profound effect on the biological properties and delivery efficacy. In a different context, the amphiphilic block polymers, showed that micelleplexes outperform polyplexes for the delivery of pDNA.^[21]^ Beside the co‐formulation approach of polyplexes, for instance with PEG groups,^[22]^ that is used to reduce their immunogenicity and to improve their biological stability, a current endeavor is to synergistically integrate different ingredients (cationic, lipophilic and hydrophilic groups) into a single vector.^[23]^

Pioneering work of our group introduced a powerful Dynamic Constitutional Strategy, combining easy synthesis and rapid screening that enable the selection of highly effective Dynamic Constitutional Frameworks‐DCFs for DNA transfection.[^6^, ^7^, ^8^, ^9^] Differently to the rational design, the DNA itself is used to self‐select an active Dynamic DNA Vector‐DyNAvector from the virtual mixture of architectures, resulting in a highly useful simplified screening process. PEGylated Squalene‐SQ has been used to form well‐defined complexes of pDNA and DyNAvectors, which composing of hydrophilic corona and hydrophobic SQ dense core used for DNA transfection^[8a]^ or enzyme activation.^[8b]^

However, only in a limited number of cases the complexation preferences among different types of nucleic acids has been investigated.^[24]^ This is also becoming important as more examples have now reported the templating role that nucleic acids play in the formation of the polyplex nanoparticles.[^10^, ^25^] For instance, it has been reported that folding of plasmid DNA – a general process called DNA condensation^[26]^ – by cationic vectors yields rod structures which size is a quantized length of the original pDNA length.^[27]^ Hence, beside the interesting challenge of fine‐tuning a (macro)molecular vector for binding and transporting a given nucleic acid, an alternative approach made possible by the recent expansion of nucleic acid therapeutics could also be to explore the complexation preferences between different types of nucleic acids in order to find the best suited application of a given compound.

Herein, we explored the complexation preferences of a library of 40 dynamic covalent frameworks (DCFs) toward different types of DNA: a circular plasmid DNA (pDNA, 5700 bp), a long double‐stranded DNA (calf thymus, ctDNA, 20000 bp) and a shorter double‐stranded DNA (salmon sperm, spDNA, 2000 bp). DNA complexation, assessed by gel electrophoresis and fluorescence displacement assays, revealed an interesting adaptive and selective complexation preference of DCFs towards different nucleic acids. The DLS and TEM characterization of the polyplex nanoparticles, confirm the observed complexation preferences.

## Results and Discussion

### Design of dynamic constitutional frameworks (DCFs)

A broad range of DCF vectors have been fabricated for the studies described here. In order to study the structure‐activity effect and the nucleic acids binding preferences, 40 DCFs were designed purposefully by using reversible amino‐carbonyl/imine chemistry^[28]^ with squalene (**SQ**), amino‐terminated polyethylene glycols (**PEG**), benzene‐1,3,5‐tricarboxaldehyde (**BTA**) and amino‐terminated positively‐charged binding groups. As a core‐center, benzene 1,3,5‐tricarboxaldehyde enables to sequentially connect the amino‐terminated squalene or polyethylene glycol segments and cationic binding sites in acetonitrile or water as solvents (see Experimental Section for details). Aldehyde‐modified squalene can act as a hydrophobic component to initiate the nano‐particle formation in aqueous environment.[^29^, ^30^]

**Scheme 1:**
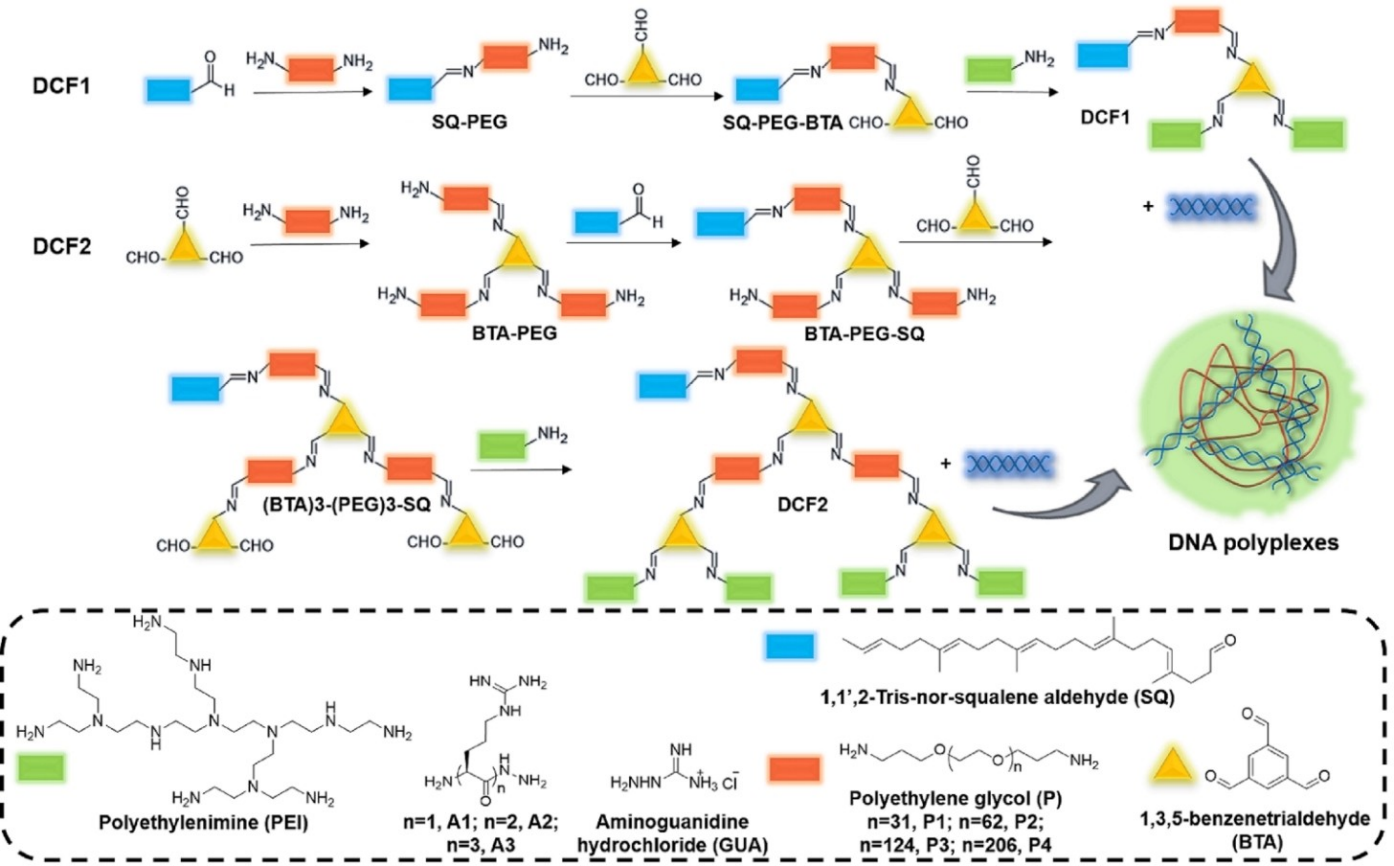
Schematic representation of dynamic constitutional frameworks (DCF1 and DCF2) and polyplexes.

Five cationic head‐groups, aminoguanidine hydrochloride (**GUA**), arginine building blocks (**A1**, **A2** and **A3**) and branched polyethyleneimine (**PEI**: bPEI800, *M*_n_∼800 g mol^−1^) play the vital role in the multivalent binding of DNA involving salt‐bridges interactions.^[31]^ The poly(ethylene glycol) bis (3‐amino propyl) terminated with different molecular weights (**P1**: *n*=31, 1500 g mol^−1^; **P2**: *n*=62, 3000 g mol^−1^; **P3**: *n*=124, 6000 g mol^−1^; **P4**: *n*=206, 10000 g mol^−1^) were used to adjust the length between the squalene hydrophobic tail and the cationic headgroups (Scheme 1). Additionally, varying the PEG length was also shown to profoundly impact the structure of the polyplex nanoparticles.^[32]^

Using the versatile dynamic constitutional synthetic methodology, two types of **DCFs**, amounting to different dendron generations, were prepared by varying the relative ratio among the four different building blocks: **DCF1** presents a simple hydrophilic segment unit at the ratio 1 : 1 : 1 : 2 of **SQ: PEG: BTA: GUA/A(1**‐**3)/bPEI800** and **DCF2** was obtained by adjusting the ratio to 1 : 3 : 3 : 4 of these components, forming a double hydrophilic segment terminated with positive charged heads. Varying the hydrophobic/ hydrophilic balance, the nature of the PEG and positive charged heads, it was expected to influence DNA complexation as well as the self‐assembly of the DCFs.

### Screening different DCFs for pDNA complexation

**Figure 1:**
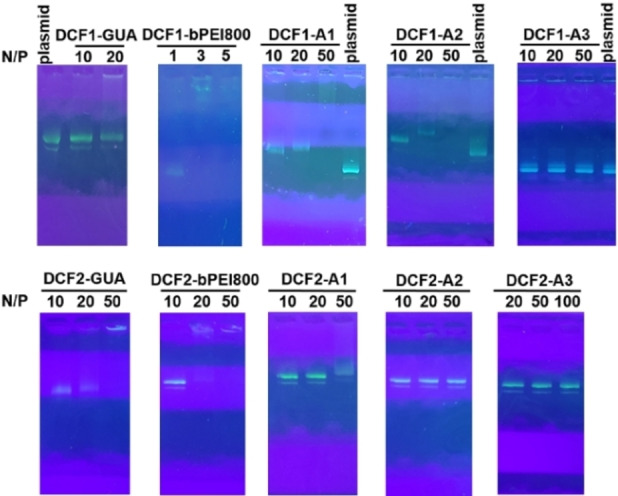
Representative gel electrophoresis results of **DCFs‐P1 (**top: **DCF1‐P1**; bottom: **DCF2‐P1)** toward pDNA with varied cationic ligands.

The binding ability of **DCFs** toward pDNA (5700 bp) in saline solution (100 mM HEPES buffer, pH 7.0, 150 mM NaCl) was first assessed by gel electrophoresis,^[33]^ monitoring the N/P ratios at which the free pDNA band disappeared due to complexation with DCFs made of the shortest **P1** PEG spacer.^[34]^ The obtained results for representative samples are summarized in Figure 1, and the N/P ratio at complete pDNA complexation are listed in Table 1. Overall, it is obvious that **DCF1‐bPEI800** present the best binding ability toward pDNA with a complete complexation achieved at N/P=3 (Figure 1), while of the corresponding **DCF2‐bPEI800** show full complexation N/P=20. Thus, in this series, increasing the valency of cationic head groups does not result in improved binding, a behavior that was previously reported using small cationic dendrons and related to differences in self‐assembly propensity.^[35]^ Replacing the PEI cationic group by **GUA** or arginine ligands decrease the efficiency of pDNA complexation in both **DCF1** and **DCF2** series. Surprisingly given the known superior binding of guanidinium groups to partake in strong salt‐bridge interactions with DNA phosphodiesters compared to the primary ammonium of lysine,[^16^, ^36^] **DCF‐A3** were found to be less active than **DCF‐A1**. Changing the short **P1** PEG spacer with the longer **P2** and **P3** has little effect on pDNA complexation. Only a weak improvement was observed when increasing the PEG length in the **DCF1** series, especially with **GUA** and **A1** headgroups (Figure S1 and Table S1), while no effect could be observed in the **DCF2** series (Figure S2 and Table S2). In conclusion, the **DCF1** architecture made of **PEI** cationic headgroup was identified as the most effective for pDNA complexation.

**Table 1:** Summary on N/P ratio of fully complexation of **DCFs‐P1** toward pDNA with varied cationic ligands.

Sample	GUA	bPEI800	A1	A2	A3
**DCF1‐P1**	>20^[b]^	3	50	∼50^[a]^	>50^[b]^
**DCF2‐P1**	50	20	>50^[b]^	>50^[b]^	>100^[b]^

[a] approximate N/P ratio at which the complexation takes place.

### Screening DNA complexation preferences of DCF‐PEI

A fluorescence displacement assay^[37]^ was then used to investigate the complexation of **DCF1‐bPEI800** and **DCF2‐bPEI800** toward different DNA varying in length: a long double‐stranded DNA (calf thymus, ctDNA, 20000 bp) and a shorter double‐stranded DNA (salmon sperm, spDNA, 2000 bp) which are both commercially‐available in quantities suited for this assay. In this ethidium bromide (EthBr) assay carried out in saline solution (100 mM HEPES buffer, pH 7.0, 150 mM NaCl), DNA complexation triggers the release of intercalated EthBr which can be detected by a decrease of fluorescence emission (*λ*_ex_ 480 nm, λ_em_ 620 nm, see Figures S3 and S4).

**Figure 2:**
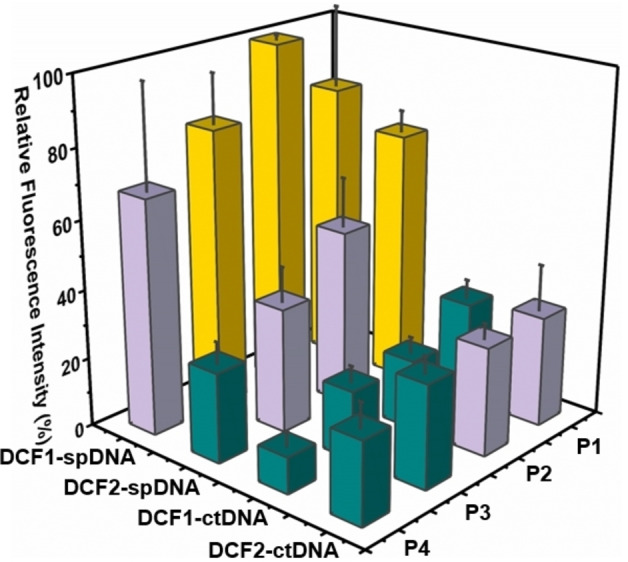
Relative fluorescence emission of **DCF1‐bPEI800** complexes and **DCF2‐bPEI800** complexes with ctDNA or spDNA at N/P=20: green 0–30 %; purple 30–70 %; yellow 70–100 %.

We performed the experiments at different N/P ratios and here describe the representative results at N/P=20 where complete pDNA complexation was observed (see above). As shown in Figure 2 which represent the relative fluorescence emission upon addition of the DCFs onto the ctDNA:EthBr complex, **DCF1‐P1‐bPEI800** and **DCF2‐P1‐bPEI800** now show a similar decrease in the fluorescence emission which indicate an identical propensity to complex ctDNA. However, while increasing the PEG spacer from **P1** to **P4** has no measurable effect on **DCF2‐bPEI800**, a constant improvement is seen with **DCF1‐bPEI800**. Complexation to the smaller spDNA appears generally weaker, with now **DCF2‐P1‐bPEI800** surpassing **DCF1‐P1‐bPEI800** (Figure 2). In this case, increasing the PEG spacer is ineffective on **DCF1‐bPEI800** but continuously improves spDNA complexation using **DCF2‐bPEI800**.

Isothermal titration calorimetry (ITC) was used as an alternative technique to cross‐check those results. Using ctDNA, binding was observed with the four ligands tested: **DCF1‐P1‐bPEI800**, **DCF1‐P4‐bPEI800**, **DCF2‐P1‐bPEI800**, and **DCF2‐P4‐bPEI800** (Table 2). In all cases, the binding stoichiometry lied around unity, which is surprising given the reduced number of positive charges in bPEI800 (ca. 16 repeating units, hence ca. 16 nitrogens) compared to ctDNA (20000 bp, hence 40000 phosphates), and the different number of bPEI800 per monomer (2 in DCF1, 4 in DCF2). Therefore, we propose that the DCFs are already organized in nanoparticles (see below), and that ITC serves here to probe the overall binding of those nanoparticles to DNA. In line with the results from the fluorescent displacement assay (Figure 2), **DCF1‐P4‐bPEI800** came out as the best complexing agent (overall *K*_d_=1.2 μM, two‐step binding process, mainly entropy‐driven).

**Table 2:** Thermodynamic parameters derived from isothermal titration calorimetry binding studies for the complexation of ctDNA and spDNA by DCFs.

	ctDNA				spDNA			
Compounds	*K*_d_ [μM]	N	ΔH [kcal mol^−1^]	−TΔS [kcal mol^−1^]	*K*_d_ [μM]	N	ΔH [kcal mol^−1^]	−TΔS [kcal mol^−1^]
**DCF1‐P1‐bPEI800**	30.8±8.3	0.9	18.4	−24.4	134±90	0.5	43.2	−48.5
**DCF1‐P4‐bPEI800**	13.6±0.2 0.085±0.016	0.9 2.6	1.2 0.2	−7.9 −9.8	n.d.			
**DCF2‐P1‐bPEI800**	2.2±0.4	0.8	−18.6	10.9	n.d.			
**DCF2‐P4‐bPEI800**	10.4±2.8	0.9	5.4	−12.2	43±10	0.8	12.2	−18.1

[a] n.d.: not determined because binding too weak.

**DCF1‐P1‐bPEI800** complexes ctDNA weaklier (*K*_d_=30.8 μM) and binding is now more entropy driven, thus pointing to the important role of the PEG spacer. In contrast, **DCF2‐P1‐bPEI800** appeared superior to **DCF2‐P4‐bPEI800** for complexing ctDNA (*K*_d_=2.2, enthalpy driven vs. 6.0 μM, entropy driven, respectively). Again, two‐steps are supposed to be involved in the binding of **DCF2‐P4‐bPEI800** to ctDNA, suggesting a different complexation mode in the presence of the longer **P4** PEG spacer. Complexation of spDNA by the four ligands tested was much weaker. No binding could be detected for **DCF1‐P4‐bPEI800** and **DCF2‐P1‐bPEI800**.

However, **DCF2‐P4‐bPEI800** indicated improved binding to spDNA compared with **DCF1‐P1‐bPEI800** (*K*_d_=43 vs. 134 μM) in agreement with fluorescence measurements. The association of spDNA by **DCF1‐P1‐bPEI800** was 4.4‐fold weaker than ctDNA (K_d_=134 vs. 30.8 μM, respectively), supporting the trend previously unraveled by the fluorescent displacement assay (Figure 2). On the other hand, complexation by **DCF2‐P4‐bPEI800** was less affected (2.4‐fold) when using spDNA as compared to ctDNA (*K*_d_=43 vs. 17.7 μM, respectively). In conclusion, **DCF1‐P4‐bPEI800** was identified using two different techniques (fluorescent displacement assay and isothermal titration calorimetry) as best suited for ctDNA complexation. As suggested above, these different complexation preferences probably originate from changes in the nanoscale organization of the polyplexes made of different types of DNA.

### Characterization of polyplex nanoparticles

Dynamic light scattering (DLS) was further used to characterize the self‐assemblies and determine the average diameter of the complexes formed by **DCFs‐P1‐bPEI800** and DNAs in saline solution (100 mM HEPES buffer, pH 7.0, 150 mM NaCl). Non‐complexed **DCF1‐P1‐bPEI800** and **DCF2‐P1‐bPEI800** form nanoparticles of, respectively, 60 nm and 91 nm size (Table 3).

**Table 3:** Representative particle size DLS characterization of the complexes formed by DCFs‐bPEI800 with PEG1500 as hydrophilic chain and ctDNA or spDNA at N/P=20 respectively, unit: nm.

Sample	DCF1‐P1‐bPEI800 size±SD [nm]	DCF2‐P1‐bPEI800 size±SD [nm]
Without DNA	60.1±0.9	90.8±1.5
Calf thymus DNA	496.1±36.8	759.7±17.3
Salmon sperm DNA	687.5±31.9	2224±47.8^[a]^

[a] Sample displaying a high polydispersity (PDI>0.8).

**Figure 3:**
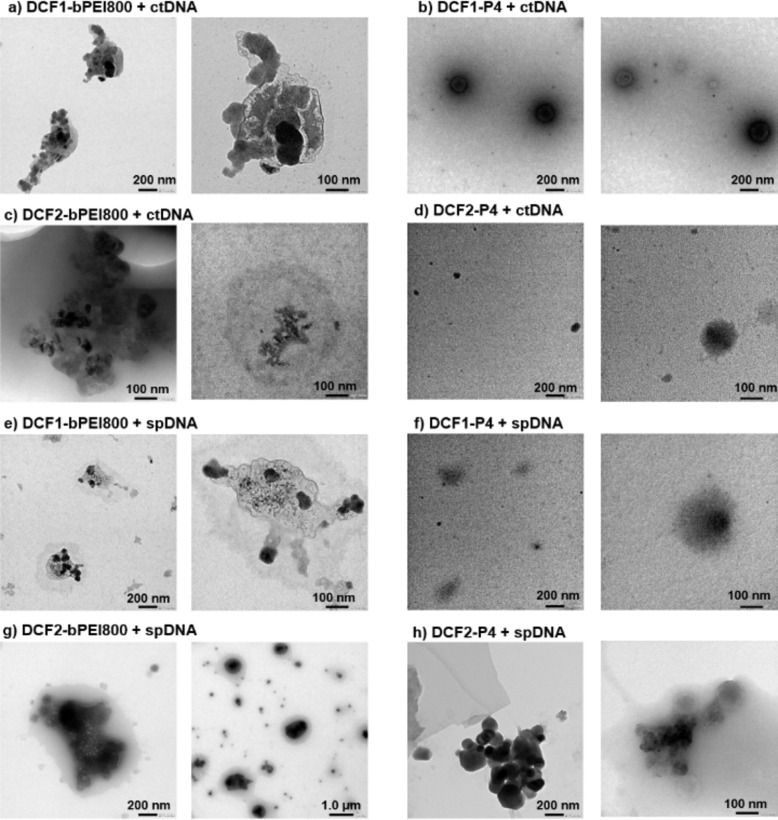
Representative TEM micrographs recorded from a) **DCF1‐P1‐bPEI800**, b) **DCF1‐P4‐bPEI800**, c) **DCF2‐P1‐bPEI800**, d) **DCF2‐P4‐bPEI800** complexes with ctDNA; and e) **DCF1‐P1‐bPEI800**, f) **DCF1‐P4‐bPEI800**, g) **DCF2‐P1‐bPEI800**, h) **DCF2‐P4‐bPEI800** with spDNA at N/P=20.

Then, the results on the DNA complexes formed at N/P=20 showed that the **DCF1** tend to form smaller complexes with ctDNA or spDNA compared to **DCF2**. Also, smaller nanoparticles are formed with the longer ctDNA compared to the shorter spDNA. In a subsequent effort to elucidate the morphological architectures of DNA complexes, Transmission electronic microscopy (TEM) was performed for the formulations **DCFs‐P1‐bPEI800** and **DCFs‐P4‐bPEI800** complexed to either ctDNA or spDNA. The apparent average sizes show the same trend as DLS but dry particle dimensions as determined by TEM are smaller (Figure 3) than the hydrodynamic diameter measured by DLS, which indicate that a hydration shell surrounds the polyplex nanoparticles in solution.

As shown in Figure 3, condensed assemblies of **DCF1‐P1‐bPEI800** and ctDNA polyplexes were present by transmission electron microscopy with a size of about 500 nm at N/P=20 (Figure 3a). On the same composition condensed assemblies of **DCF1‐P4‐bPEI800** and ctDNA polyplexes form highly uniform spherical aggregates of about 200 nm diameter (Figure 3b). Moreover, it is worth to note that ctDNA was intertwined with several irregular particles in **DCF1‐P1‐bPEI800**/ctDNA polyplex while being encapsulated in spherical vesicles of **DCF1‐P4‐bPEI800**/ctDNA polyplex, revealing the binding of **DCFs** particles to the folded double strands adaptively. Similarly, the polyplexes of DCF2‐ctDNA was crowded with more dendritic but smaller **DCF2‐P1‐bPEI800**/ctDNA polyplex particles (Figure 3c, dense electronic regions), surrounding by a dynamically ordered hydration layer or smaller compact **DCF2‐P4‐bPEI800**/ctDNA polyplex particles of about 100 nm diameter (Figure 3d).

The binding situation slightly changed, when a shorter double‐strands (spDNA) was involved to complex with **DCFs**. Similarly, the aggregated crowding phenomenon happened also to spDNA with a few encapsulated **DCF1‐P1‐bPEI800** inside and the hanged ones around the side (Figure 3e). **DCF1‐P4‐bPEI800** and spDNA polyplexes still form highly uniform 200 nm spherical aggregates (Figure 3f). The dense core was more obvious with regarding to the **DCF2‐P1‐bPEI800** spDNA (Figure 3g) and **DCF2‐P4‐bPEI800** spDNA (Figure 3h) samples, occupying most of the complexed area with also the hydration layer surrounding. An average size of around 1 μm was observed from the obtained polyplexes DCF2‐spDNA, showing a hydrated layer but bigger diameters than that of DCF2‐ctDNA. It indicated that the multivalent **DCFs** tend to aggregate with shorter double strands to a certain extent.

This discrete void in all present samples would be attributed to the assembling hydrophilic polycations which enable to bind electrostatically with DNA, while excessive cationic bPEI800 makes the difference on providing a hydrated isolation. In terms of the shape and nanoscale size of **DCFs** from TEM, we know that that hydrophobic squalene chain played the role in controlling the compact size of complexes, achieving the optimal DNA‐ligand assembly. Increasing the PEG‐chain length may change the structure of the polyplexes, as shown in by morphological aggregation in dendritic structures of **P1**‐type DCFs and compact spheres of **P4**‐type DCFs Comparing the binding efficiency, the TEM results confirm that best binding is obtained for a narrow polydispersity observed in **DCF1,2‐P4‐bPEI800**, with optimal diameter of between 100–200 nm, whilst higher polydispersity and diameter of polyplexes **DCF1,2‐P1‐bPEI800** gave poorer binding values. We know from previous studies, that PEG crowding in the package of DNAs play an important role as a high overlapping of higher PEG chains is relevant for a better transfection.^[31]^

Structure‐activity effect can be displayed on DNA/DCFs binding by comparing variant self‐assembled polycations. As a shape‐ persistent polyanion, ctDNA and spDNA would select **DCFs** according to their inherent architectures, being selectively complexed with these **DCFs**. The reversible covalent linkages among building blocks provide variation for assembly with nucleic acids adaptively, this enabled nucleic acids to recognize the optimal ligands from intricate dynamic systems. In the case of ctDNA, it tends to be complexed at N/P20, no matter by the virtue of intertwinement with **DCF1** or simple multivalent encapsulation with **DCF2**. Complexing efficiency was not as obvious as ctDNA when it came to spDNA, whereas it tends to entangle with **DCF2**, but interacted with **DCF1** through electrostatic attraction on surfaces. Thus, we can assume that longer nucleic acids match ligands for complexing easily, and the apparent preference to optimal **DCFs** is attributed to adaptive recognition and intertwined assembly regardless of length of nucleic acids.

## Conclusion

We reported here the preparation of a library of 40 dynamic constitutional frameworks varying hydrophilic/hydrophobic balance, number and nature of cationic headgroups, and length of PEG spacers. Marked differences in terms of DNA complexation efficiency were observed between the different DCFs. While the PEI cationic head group was showed to be best for promoting DNA complexation, the lower generation dendrons **DCF1** were found more potent for pDNA complexation than **DCF2**. However, exploring the complexation of ctDNA and spDNA revealed different behaviors. For instance, carefully looking at the weaker effect of the PEG spacers showed that **DCF1‐bPEI800** series was best suited for ctDNA complexation, while **DCF2‐bPEI800** series was best for complexing the shorter spDNA. Finally, DLS and TEM analyses using P4 PEG spacer suggest that condensation into nanoparticles is more effective, which could be the result of hydrophobic collapse best promoted using longer P4 PEG. **DCF1‐P4‐bPEI800** was identified as best suited for ctDNA complexation, while **DCF2‐P4‐bPEI800** was best for complexing the shorter spDNA. Overall, these results show that DNA complexation can not only be fine‐tuned by molecular engineering of the vector, but also by the nature of the DNA that acts as a template in the formation of the final polyplex nanoparticle. More generally, this work points out to the need to consider the nucleic acid templating role when considering evolutionary approaches for making therapeutic polyplexes.^[38]^

## Experimental Section

**Materials and methods**: All reagents were bought from Sigma Aldrich and used without further purification. ^1^H NMR spectra were recorded on a 400 MHz Bruker spectrometer, and peaks are referenced in ppm with respect to the residual solvent peak. 1,1’,2‐Tris‐nor‐squalene aldehyde (**SQ**) was synthesized through a 3‐step reaction as previously reported.^[30]^

**Gel electrophoresis**: Gel retardation assays were carried out by mixing plasmid DNA (5700 bp, 100 ng) and **DCFs** in HEPES buffer (100 mM, pH 7.0, 10 μM EDTA, 150 mM NaCl) to a total volume of 12 μL with 2 μL of Blue 6× loading dye (Fisher Scientific), incubating for 30 minutes. All samples were run on a 0.7 % agarose gel (50 V) for 20 minutes in 0.5× TBE buffer (20 mM Tris‐borate/0.5 mM EDTA, pH 8.2) and visualized with SYBER Safe (Life Technologies).

**Fluorescence displacement assay**: The complexation ability of **DCFs** was evaluated by a fluorescence displacement assay by using a spectrofluorometer (SAFAS Xenius), with calf thymus DNA (ctDNA, 13.3 μg/mL) and salmon sperm DNA (spDNA, 13.3 μg/mL) by using ethidium bromide (5 μM). The binding behavior was evaluated by monitoring the fluorescence emission at 620 nm and excited at 480 nm.

All samples were prepared in HEPES buffer (100 mM, pH 7.0). The relative fluorescence emission was calculated as following: Relative fluorescence emission=(*I*_2_−*I*_0_)/(*I*_1_−*I*_0_), where *I*_2_ represents the fluorescence emission of **DCFs**‐complexed DNA and ethidium bromide; *I*_1_ represents the fluorescence emission of DNA and ethidium bromide; *I*_0_ represents the fluorescence emission of ethidium bromide.

**Isothermal titration calorimetry**: The thermodynamic binding parameters were recorded at 25 °C on a MicroCal/Malvern PEAK‐ITC (Malvern Panalytics) instrument. The titration cell was filled with a solution of ctDNA or spDNA (25–50 μM), and the syringe was loaded with a solution of DCFs. For each experiment, a series of injections of ligand (0.3–1 mM) from a rotating syringe (speed 750 rpm) were made into the thermostatic cell (initial delay of 60 s, duration of 2 s and spacing of 120 s). Control experiments were performed by adding the ligand solution to the cell containing the HEPES buffer. The corrected ITC titrations were processed using the MicroCal Origin software.

**Dynamic light scattering (DLS)**: The size of polyplexes were determined by DLS from measurements recorded on Zetasizer Nano (Malvern) using solutions prepared in HEPES buffer solution with the corresponding nucleic acids at N/P=20. Measurements were performed after incubating **DCFs** and DNA for 30 minutes at 25 °C.

**Transmission electron microscopy (TEM)**: TEM images were obtained by using a JEM 1400+ electron microscopy: 20 μL of sample solution was dropped on a carbon coated copper grid and dried at room temperature. Then the sample was observed at a 120 kV acceleration voltage.

**Preparation of DCF1**. **DCF1** was obtained through the following steps:

- **SQ‐PEG**: 1,1’,2‐Tris‐nor‐squalene aldehyde (104 mg, 0.27 mmol, 1 equiv.), and poly(ethylene glycol) diamine **PEG** (**P1**: **PEG1500**, 445 mg, 0.297 mmol, 1.1 equiv.; **P2: PEG3000**, 891 mg, 0.297 mmol, 1.1 equiv.; **P3**: **PEG6000**, 1782 mg, 0.297 mmol, 1.1 equiv.; **P4: PEG10 K**, 2970 mg, 0.297 mmol, 1.1 equiv.) were mixed in acetonitrile at 80 °C for 24 h. The resulting product (**SQ‐PEG**) was verified through ^1^H NMR and stocked for next step.
- **SQ‐PEG‐BTA**: A solution of 1.3.5‐benzenetrialdehyde (43.4 mg, 0.27 mmol, 1 equiv.) in acetonitrile was added to a solution of **SQ‐PEG** (0.27 mmol, 1 equiv.) at 80 °C for 24 h. The reaction was monitored through ^1^H NMR until the equilibrium was reached and the resulting **SQ‐PEG‐BTA** product was stocked in milli‐Q water at 10 mM of concentration.
- **DCF1**: Positively‐charged compounds, arginine building blocks (1 mL of 20 mM aqueous solution, 2 equiv.), aminoguanidine hydrochloride (1 mL of 20 mM aqueous solution, 2 equiv.), **bPEI800** (1 mL of 20 mM aqueous solution, 2 equiv.) were added to the above intermediary solution, respectively, obtaining the corresponding **DCF1** stock solution (5 mM). Equilibrium was monitored through ^1^H NMR.

**Preparation of DCF2**. **DCF2** was obtained through the following steps:

- **BTA‐PEG**: Poly(ethylene glycol) diamine **PEG** (**PEG1500**, 225 mg, 0.15 mmol, 3 equiv.; **PEG3000**, 450 mg, 0.15 mmol, 3 equiv.; **PEG6000**, 900 mg, 0.15 mmol, 3 equiv.; **PEG10 K**, 1500 mg, 0.15 mmol, 3 equiv.) and 1.3.5‐benzenetrialdehyde (8.1 mg, 0.05 mmol, 1 equiv.) were mixed in acetonitrile at 80 °C for 24 h. The resulting **BTA‐PEG** was verified through ^1^H NMR.
- **BTA‐PEG‐SQ**: A solution of 1,1’,2‐Tris‐nor‐squalene aldehyde (19 mg, 0.05 mmol, 1 equiv.) in acetonitrile was added to the above **BTA‐PEG** solution, reacting at 80 °C for 24 h. The resulting **BTA‐PEG‐SQ** was monitored through ^1^H NMR until the equilibrium.
- **(BTA)3‐(PEG)3‐SQ**: 1.3.5‐benzenetrialdehyde (16.2 mg, 0.10 mmol, 2 equiv.) was added to the above intermediary aqueous solution and the equilibrium was monitored through ^1^H NMR. And the resulting **intermediary product** was stocked in milli‐Q water at 10 mM of concentration.
- **DCF2**: Positively‐charged compounds, arginine building blocks (2 mL of 20 mM aqueous solution, 4 equiv.), aminoguanidine hydrochloride (2 mL of 20 mM aqueous solution, 4 equiv.), **bPEI800** (2 mL of 20 mM aqueous solution, 4 equiv.) were added to the above intermediary solution respectively, obtaining the corresponding **DCF2** stock solution (5 mM). Equilibrium was monitored through ^1^H NMR.

## Conflict of interest

The authors declare no conflict of interest.

1

## Supporting information

As a service to our authors and readers, this journal provides supporting information supplied by the authors. Such materials are peer reviewed and may be re‐organized for online delivery, but are not copy‐edited or typeset. Technical support issues arising from supporting information (other than missing files) should be addressed to the authors.

Supporting Information

## Data Availability

The data that support the findings of this study are available from the corresponding author upon reasonable request.

## References

[1a] L. Peng , E. Wagner , Biomacromolecules 2019, 20, 3613–3626; 31497946 10.1021/acs.biomac.9b00999

[1b] H. Yin , R. L. Kanasty , A. A. Eltoukhy , A. J. Vegas , J. R. Dorkin , D. G. Anderson , Nat. Rev. Genet. 2014, 15, 541–555. 25022906 10.1038/nrg3763

[2a] N. Chaudhary , D. Weissman , K. A. Whitehead , Nat. Rev. Drug Discovery 2021, 20, 817–838; 34433919 10.1038/s41573-021-00283-5 PMC8386155

[2b] E. Dolgin , Nature 2021, 597, 318–324. 34522017 10.1038/d41586-021-02483-w

[3a] U. Lächelt , E. Wagner , Chem. Rev. 2015, 115, 11043–11078; 25872804 10.1021/cr5006793

[3b] R. Kumar , C. F. Santa Chalarca , M. R. Bockman , C. van Bruggen , C. J. Grimme , R. J. Dalal , M. G. Hanson , J. K. Hexum , T. M. Reineke , Chem. Rev. 2021, 121(18), 11527–11652. 33939409 10.1021/acs.chemrev.0c00997

[4] O. Boussif , F. Lezoualc′h , M. A. Zanta , M. D. Mergny , D. Scherman , B. Demeneix , J.-P. Behr , Proc. Nat. Acad. Sci. 1995, 92, 7297–7301. 7638184 10.1073/pnas.92.16.7297 PMC41326

[5a] E. I. Geihe , C. B. Cooley , J. R. Simon , M. K. Kiesewetter , J. A. Edward , R. P. Hickerson , R. L. Kaspar , J. L. Hedrick , R. M. Waymouth , P. A. Wender , Proc. Nat. Acad. Sci. 2012, 109, 13171–13176; 22847412 10.1073/pnas.1211361109 PMC3421195

[5b] R. Qi , S. Wu , H. Xiao , L. Yan , W. Li , X. Hu , Y. Huang , X. Jing , J. Mater. Chem. 2012, 22, 18915–18922;

[5c] K. Klemm , M. Radić Stojković , G. Horvat , V. Tomišić , I. Piantanida , C. Schmuck , Chemistry 2012, 18, 1352–1363; 22213523 10.1002/chem.201101544

[5d] T.-i. Kim , M. Lee , S. W. Kim , Biomaterials 2010, 31, 1798–1804; 19854504 10.1016/j.biomaterials.2009.10.034 PMC2813999

[5e] T. A. Theodossiou , A. Pantos , I. Tsogas , C. M. Paleos , ChemMedChem 2008, 3, 1635–1643; 18985650 10.1002/cmdc.200800190

[5f] L.-A. Tziveleka , A.-M. G. Psarra , D. Tsiourvas , C. M. Paleos , J. Control Release 2007, 117, 137–146. 17156881 10.1016/j.jconrel.2006.10.019

[6a] R. Catana , M. Barboiu , I. Moleavin , L. Clima , A. Rotaru , E.-L. Ursu , M. Pinteala , Chem. Commun. 2015, 51, 2021–2024; 10.1039/c4cc07525k 25500992

[6b] I.-A. Turin-Moleavin , F. Doroftei , A. Coroaba , D. Peptanariu , M. Pinteala , A. Salic , M. Barboiu , Org. Biomol. Chem. 2015, 13, 9005–9011. 26171592 10.1039/c5ob01315a

[7] L. Marin , D. Ailincai , M. Calin , D. Stan , C. A. Constantinescu , L. Ursu , F. Doroftei , M. Pinteala , B. C. Simionescu , M. Barboiu , ACS Biomater. Sci. Eng. 2016, 2, 104–111. 33418648 10.1021/acsbiomaterials.5b00423

[8a] L. Clima , D. Peptanariu , M. Pinteala , A. Salic , M. Barboiu , Chem. Commun. 2015, 51, 17529–17531; 10.1039/c5cc06715d 26469153

[8b] D.-D. Su , K. Aissou , Y. Zhang , V. Gervais , S. Ulrich , M. Barboiu , Catal. Sci. Technol. 2022, 12, 3094–3101.

[9a] Y. Zhang , Y. C. Qi , S. Ulrich , M. Barboiu , O. Ramström , Mater. Chem. Front. 2020, 4, 489–506; 33791102 10.1039/c9qm00598f PMC8009197

[9b] D. Su , M. Coste , A. Diaconu , M. Barboiu , S. Ulrich , J. Mater. Chem. B 2020, 8, 9385–9403; 33048107 10.1039/d0tb01836h

[9c] C. Bouillon , Y. Bessin , F. Poncet , M. Gary-Bobo , P. Dumy , M. Barboiu , N. Bettache , S. Ulrich , J. Mater. Chem. B 2018, 6, 7239–7246; 32254636 10.1039/c8tb01278d

[9d] C. Bouillon , D. Paolantoni , J. C. Rote , Y. Bessin , L. W. Peterson , P. Dumy , S. Ulrich , Chem. – Eur. J. 2014, 20, 14705–14714. 25251569 10.1002/chem.201403695

[10] N. Laroui , M. Coste , D. Su , L. Ali , Y. Bessin , M. Barboiu , M. Gary-Bobo , N. Bettache , S. Ulrich , Angew. Chem. Int. Ed. 2020, 11, 5783–5787. 10.1002/anie.202014066 33289957

[11a] E. Bartolami , Y. Bessin , N. Bettache , M. Gary-Bobo , M. Garcia , P. Dumy , S. Ulrich , Org. Biomol. Chem. 2015, 13, 9427–9438; 26248061 10.1039/c5ob01404b

[11b] E. Bartolami , Y. Bessin , V. Gervais , P. Dumy , S. Ulrich , Angew. Chem. Int. Ed. 2015, 54, 10183–10187; 10.1002/anie.201504047 26177835

[11c] I. Nakase , H. Akita , K. Kogure , A. Gräslund , U. Langel , H. Harashima , S. Futaki , Acc. Chem. Res. 2012, 45, 1132–1139; 22208383 10.1021/ar200256e

[11d] P. Boisguérin , S. Deshayes , M. J. Gait , L. O′Donovan , C. Godfrey , C. A. Betts , M. J. A. Wood , B. Lebleu , Adv. Drug Delivery Rev. 2015, 87, 52–67; 10.1016/j.addr.2015.02.008 PMC7102600 25747758

[11e] J. Hoyer , I. Neundorf , Acc. Chem. Res. 2012, 45, 1048–1056; 22455499 10.1021/ar2002304

[11f] F. S. Hassane , A. F. Saleh , R. Abes , M. J. Gait , B. Lebleu , Cell. Mol. Life Sci. 2010, 67, 715–726. 19898741 10.1007/s00018-009-0186-0 PMC11115801

[12a] I. Lostalé-Seijo , J. Montenegro , Nat. Chem. Rev. 2018, 2, 258–277;

[12b] R. Mogaki , P. K. Hashim , K. Okuro , T. Aida , Chem. Soc. Rev. 2017, 46, 6480–6491; 29034942 10.1039/c7cs00647k

[12c] X. Chen , K. Xu , J. Yu , X. Zhao , Q. Zhang , Y. Zhang , Y. Cheng , Biomater. Sci. 2020, 8, 6301–6308; 33020778 10.1039/d0bm01231a

[12d] Z. Tan , Y. K. Dhande , T. M. Reineke , Bioconjugate Chem. 2017, 28, 2985–2997. 10.1021/acs.bioconjchem.7b00598 29193962

[13a] K. Petkau-Milroy , M. H. Sonntag , A. H. A. M. van Onzen , L. Brunsveld , J. Am. Chem. Soc. 2012, 134, 8086–8089 ; 22540882 10.1021/ja3029075

[13b] R. Martinent , D. Du , J. López-Andarias , N. Sakai , S. Matile , ChemBioChem. 2021, 22, 253–259 ; 32975867 10.1002/cbic.202000630

[13c] H. Fernández-Caro , I. Lostalé-Seijo , M. Martínez-Calvo , J. Mosquera , J. L. Mascareñas , J. Montenegro , Chem. Sci. 2019, 10, 8930–8938 ; 32110291 10.1039/c9sc02906k PMC7017865

[13d] S. van Dun , J. Schill , L.-G. Milroy , L. Brunsveld , Chem. Eur. J. 2018, 24, 16445–16451; 30155918 10.1002/chem.201804045 PMC6282950

[13e] P. Morelli , E. Bartolami , N. Sakai , S. Matile , Helv. Chim. Acta. 2018, 101, e1700266;

[13f] E. Bartolami , C. Bouillon , P. Dumy , S. Ulrich , Chem. Commun. 2016, 52, 4257–4273. 10.1039/c5cc09715k 26842769

[14] C. O. Mellet , J. M. G. Fernandez , J. M. Benito , Chem. Soc. Rev. 2011, 40, 1586–1608. 21042619 10.1039/c0cs00019a

[15] L. Gallego-Yerga , M. Lomazzi , V. Franceschi , F. Sansone , C. Ortiz Mellet , G. Donofrio , A. Casnati , J. M. García Fernández , Org. Biomol. Chem. 2015, 13, 1708–1723. 25474077 10.1039/c4ob02204a

[16] C. Bienvenu , Á. Martínez , J. L. Jiménez Blanco , C. Di Giorgio , P. Vierling , C. Ortiz Mellet , J. Defaye , J. M. García Fernández , Org. Biomol. Chem. 2012, 10, 5570–5581. 22733369 10.1039/c2ob25786f

[17] A. Méndez-Ardoy , N. Guilloteau , C. Di Giorgio , P. Vierling , F. Santoyo-González , C. Ortiz Mellet , J. M. García Fernández , J. Org. Chem. 2011, 76, 5882–5894. 21707102 10.1021/jo2007785

[18a] L. Gallego-Yerga , M. J. González-Álvarez , N. Mayordomo , F. Santoyo-González , J. M. Benito , C. Ortiz Mellet , F. Mendicuti , J. M. García Fernández , Chemistry 2014, 20, 6622–6627; 24740814 10.1002/chem.201402026

[18b] L. Gallego-Yerga , L. Blanco-Fernández , K. Urbiola , T. Carmona , G. Marcelo , J. M. Benito , F. Mendicuti , C. Tros de Ilarduya , C. Ortiz Mellet , J. M. García Fernández , Chemistry 2015, 21, 12093–12104. 26184887 10.1002/chem.201501678

[19a] L. Gallego-Yerga , J. M. Benito , L. Blanco-Fernandez , M. Martinez-Negro , I. Velaz , E. Aicart , E. Junquera , C. O. Mellet , C. T. d Ilarduya , J. M. G. Fernandez , Chem. Eur. J. 2018, 24, 3825–3835; 29341305 10.1002/chem.201705723

[19b] T. Neva , A. I. Carbajo-Gordillo , J. M. Benito , H. Lana , G. Marcelo , C. O. Mellet , C. T. d Ilarduya , F. Mendicuti , J. M. G. Fernandez , Chem. Eur. J. 2020, 26, 15259–15269. 32710799 10.1002/chem.202002951

[20] A. I. Carbajo-Gordillo , M. González-Cuesta , J. L. Jiménez Blanco , J. M. Benito , M. L. Santana-Armas , T. Carmona , C. Di Giorgio , C. Przybylski , C. Ortiz Mellet , C. Tros de Ilarduya , et al., Chemistry 2021, 27, 9429–9438. 33882160 10.1002/chem.202100832 PMC8361672

[21] Z. Tan , Y. Jiang , W. Zhang , L. Karls , T. P. Lodge , T. M. Reineke , J. Am. Chem. Soc. 2019, 141, 15804–15817. 31553590 10.1021/jacs.9b06218

[22] M. Harada-Shiba , K. Yamauchi , A. Harada , I. Takamisawa , K. Shimokado , K. Kataoka , Gene Ther. 2002, 9, 407–414. 11960317 10.1038/sj.gt.3301665

[23a] D. Zhang , E. N. Atochina-Vasserman , D. S. Maurya , N. Huang , Q. Xiao , N. Ona , M. Liu , H. Shahnawaz , H. Ni , K. Kim , et al., J. Am. Chem. Soc. 2021, 143, 12315–12327; 34324336 10.1021/jacs.1c05813

[23b] D. Zhang , E. N. Atochina-Vasserman , D. S. Maurya , M. Liu , Q. Xiao , J. Lu , G. Lauri , N. Ona , E. K. Reagan , H. Ni , et al., J. Am. Chem. Soc. 2021, 143, 17975–17982; 34672554 10.1021/jacs.1c09585

[23c] D. Zhang , E. N. Atochina-Vasserman , D. S. Maurya , N. Huang , Q. Xiao , N. Ona , M. Liu , H. Shahnawaz , H. Ni , K. Kim , et al., J. Am. Chem. Soc. 2021, 143, 12315–12327. 34324336 10.1021/jacs.1c05813

[24a] Y. Shi , H.-J. Schneider , J. Chem. Soc.-Perkin Trans. 1999, 2, 1797–1803;

[24b] W. C. Tse , D. L. Boger , Chem. Biol. 2004, 11, 1607–1617; 15610844 10.1016/j.chembiol.2003.08.012

[24c] N. Rawat , P. Biswas , Phys. Chem. Chem. Phys. 2011, 13, 9632–9643. 21483987 10.1039/c1cp00027f

[25a] E. Bartolami , C. Bouillon , P. Dumy , S. Ulrich , Chem. Commun. 2016, 52, 4257–4273; 10.1039/c5cc09715k 26842769

[25b] P. K. Hashim , K. Okuro , S. Sasaki , Y. Hoashi , T. Aida , J. Am. Chem. Soc. 2015, 137, 15608–15611; 26648391 10.1021/jacs.5b08948

[25c] M. Surin , S. Ulrich , ChemistryOpen 2020, 9, 480–498; 32328404 10.1002/open.202000013 PMC7175023

[25d] P. Evenou , J. Rossignol , G. Pembouong , A. Gothland , D. Colesnic , R. Barbeyron , S. Rudiuk , A.-G. Marcelin , M. Ménand , D. Baigl , et al., Angew. Chem. Int. Ed. 2018, 57, 7753–7758; 10.1002/anie.201802550 29693753

[25e] X. Liu , J. Zhou , T. Yu , C. Chen , Q. Cheng , K. Sengupta , Y. Huang , H. Li , C. Liu , Y. Wang , et al., Angew. Chem. Int. Ed. 2014, 53, 11822–11827; 10.1002/anie.201406764 PMC4485617 25219970

[25f] J. Zhou , L. Sun , L. Wang , Y. Liu , J. Li , J. Li , J. Li , H. Yang , Angew. Chem. Int. Ed. 2019, 58, 5236–5240; 10.1002/anie.201813665 30809927

[26] A. Estévez-Torres , D. Baigl , Soft Matter 2011, 7, 6746–6756.

[27] K. Osada , H. Oshima , D. Kobayashi , M. Doi , M. Enoki , Y. Yamasaki , K. Kataoka , J. Am. Chem. Soc. 2010, 132, 12343–12348. 20712309 10.1021/ja102739b

[28a] Y. Zhang , E. Petit , M. Barboiu , ChemPlusChem 2018, 83, 354–360; 31957369 10.1002/cplu.201800081

[28b] Y. Zhang , C. T. Supuran , M. Barboiu , Chemistry 2018, 24, 715–720. 29119623 10.1002/chem.201704605

[29a] D. Desmaële , R. Gref , P. Couvreur , J. Controlled Release 2012, 161, 609–618; 10.1016/j.jconrel.2011.07.038 21840355

[29b] A. Babič , S. Pascal , R. Duwald , D. Moreau , J. Lacour , E. Allémann , Adv. Funct. Mater. 2017, 27, 1701839.

[30] A. Maksimenko , F. Dosio , J. Mougin , A. Ferrero , S. Wack , L. H. Reddy , A.-A. Weyn , E. Lepeltier , C. Bourgaux , B. Stella , et al., Proc. Nat. Acad. Sci. 2014, 111, E217–226. 24385587 10.1073/pnas.1313459110 PMC3896189

[31a] C. L. Yu , E. Tan , Y. Y. Xu , J. Lv , Y. Y. Cheng , Bioconjugate Chem. 2019, 30, 413–417; 10.1021/acs.bioconjchem.8b00753 30383369

[31b] Z. H. Chen , Z. Y. Lv , Y. F. Sun , Z. G. Chi , G. Y. Qing , J. Mater. Chem. B 2020, 8, 2951–2973. 32159205 10.1039/c9tb02271f

[32] K. M. Takeda , K. Osada , T. A. Tockary , A. Dirisala , Q. Chen , K. Kataoka , Biomacromolecules 2017, 18, 36–43. 27990798 10.1021/acs.biomac.6b01247

[33] L. E. Fechner , B. Albanyan , V. M. P. Vieira , E. Laurini , P. Posocco , S. Pricl , D. K. Smith , Chem. Sci. 2016, 7, 4653–4659. 30155113 10.1039/c5sc04801j PMC6013769

[34] C. M. Uritu , C. D. Varganici , L. Ursu , A. Coroaba , A. Nicolescu , A. I. Dascalu , D. Peptanariu , D. Stan , C. A. Constantinescu , V. Simion , et al., J. Mater. Chem. B 2015, 3, 2433–2446. 32262120 10.1039/c4tb02040e

[35a] P. Posocco , S. Pricl , S. Jones , A. Barnard , D. K. Smith , Chem. Sci. 2010, 1, 393–404;

[35b] N. Shah , R. J. Steptoe , H. S. Parekh , J. Pept. Sci. 2011, 17, 470–478. 21351322 10.1002/psc.1347

[36] A. Mann , G. Thakur , V. Shukla , A. K. Singh , R. Khanduri , R. Naik , Y. Jiang , N. Kalra , B. S. Dwarakanath , U. Langel , et al., Mol. Pharm. 2011, 8, 1729–1741. 21780847 10.1021/mp2000814

[37] D. L. Boger , B. E. Fink , S. R. Brunette , W. C. Tse , M. P. Hedrick , J. Am. Chem. Soc. 2001, 123, 5878–5891. 11414820 10.1021/ja010041a

[38] F. Freitag , E. Wagner , Adv. Drug Delivery Rev. 2021, 168, 30–54. 10.1016/j.addr.2020.03.005 32246984

