# Supplementary material for: Complexation Preferences of Dynamic Constitutional Frameworks as Adaptive Gene Vectors
Source: Chemistry. 2022 Dec 19;29(8):e202203062. doi: 10.1002/chem.202203062 (PMC10108089; doi:10.1002/chem.202203062)
Supplement: Supplementary file 1 — Supporting Information [file CHEM-29-0-s001.pdf]

Chemistry–A European Journal

Supporting Information

Complexation Preferences of Dynamic Constitutional Frameworks as Adaptive Gene Vectors

Dan-Dan Su, Virginie Gervais, Sébastien Ulrich,* and Mihail Barboiu*

Content

1. Gel Electrophoresis assay 2
2. Fluorescence Displacement Assay 3
3. Isothermal titration calorimetry 4
4. Synthesis of Arginine building blocks (A1, A2, A3) 4
5. Preparation of dynamic constitutional frameworks 8
5.1 Synthesis of SQ-PEG 9
5.2 Synthesis of SQ-PEG-BTA 9
5.3 Preparation of DCF1 9
5.4 Synthesis of BTA-PEG 11
5.5 Synthesis of BTA-PEG-SQ 12
5.6 Synthesis of (BTA)3-(PEG)3-SQ 12
5.7 Preparation of DCF2 12
6. References 13

1. Gel Electrophoresis assay

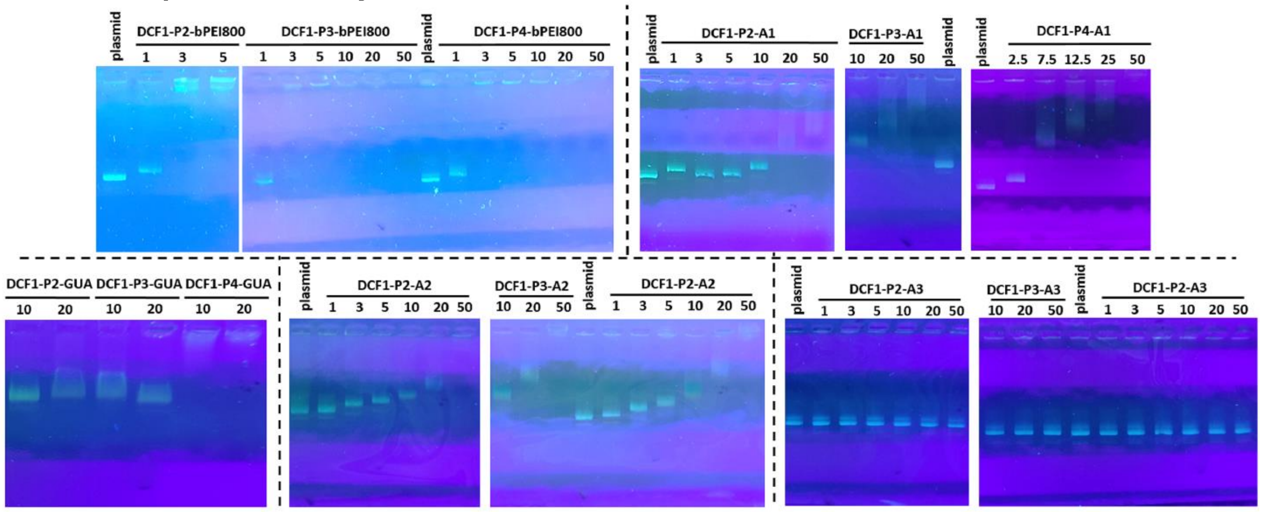

Figure S1. Gel electrophoresis results of **DCF1** with varied PEG chains (**P2, P3, P4**) and cationic ligands (**bPEI800, GUA, A1, A2, A3**) toward pDNA. For the position of the plasmid control in the DCF1-Gua gel, please refer to **Figure 1** – both were performed in the same gel electrophoresis.

Table S1. Summary of **Figure S1** on N/P ratio of complete complexation of **DCF1** toward pDNA.

Sample	GUA	bPEI800	A1	A2	A3
**DCF1-P2**	＞20[b]	3	20	10-50[a]	＞50[b]
**DCF1-P3**	＞20[b]	3	10-50[a]	10-50[a]	＞50[b]
**DCF1-P4**	10	3	2.5-25[a]	3-50[a]	＞50[b]

[a] N/P ratio range at which the complexation was observed.

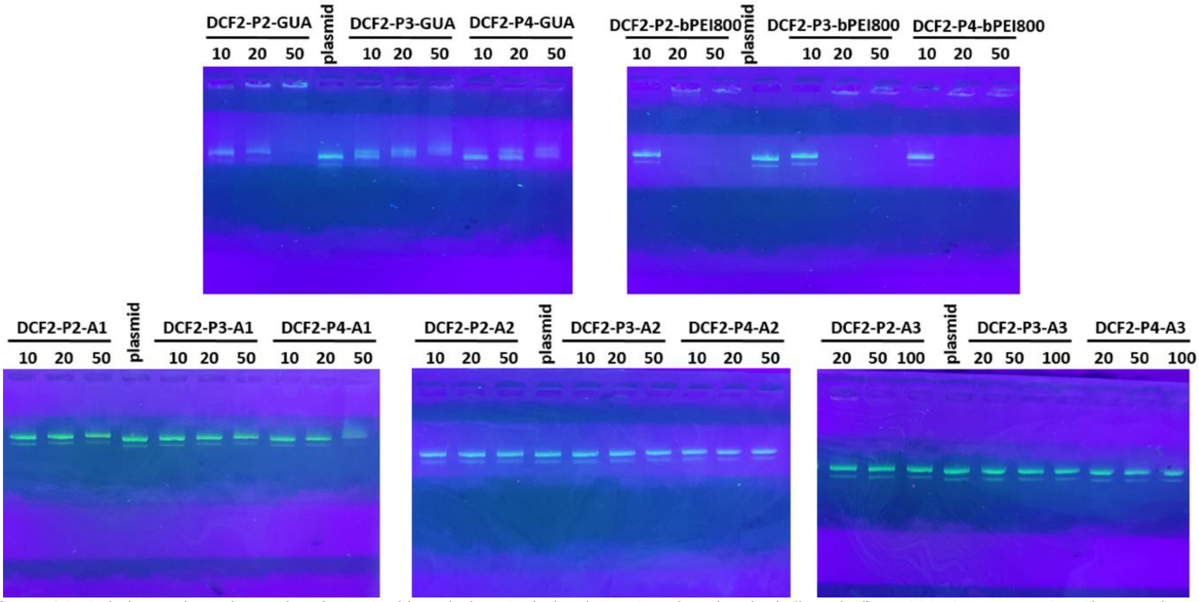

Figure S2. Gel electrophoresis results of **DCF2** with varied PEG chains (**P2, P3, P4**) and cationic ligands (**bPEI800, GUA, A1, A2, A3**) toward pDNA.

Table S2. Summary of **Figure S2** on N/P ratio of complete complexation of **DCF2** toward pDNA.

Sample	GUA	bPEI800	A1	A2	A3
**DCF2-P2**	50	20	＞50[a]	＞50[a]	＞100[a]
**DCF2-P3**	＞50[a]	20	＞50[a]	＞50[a]	＞100[a]
**DCF2-P4**	＞50[a]	20	＞50[a]	＞50[a]	＞100[a]

2. Fluorescence Displacement Assay

The excitation wavenumber was determined through a full scanning of UV-vis spectra of ethidium bromide in HEPES buffer, and the maximum emission wavenumber was obtained via the fluorescence displacement assay (**Figure S3 and S4**). As shown in Figure S3a, the concentration of ethidium bromide (EthBr) was varied from 5 μM to 100 μM for full scanning, and fixed at 5 μM for complexing with ctDNA (13.3 μg/mL), at N/P=20.

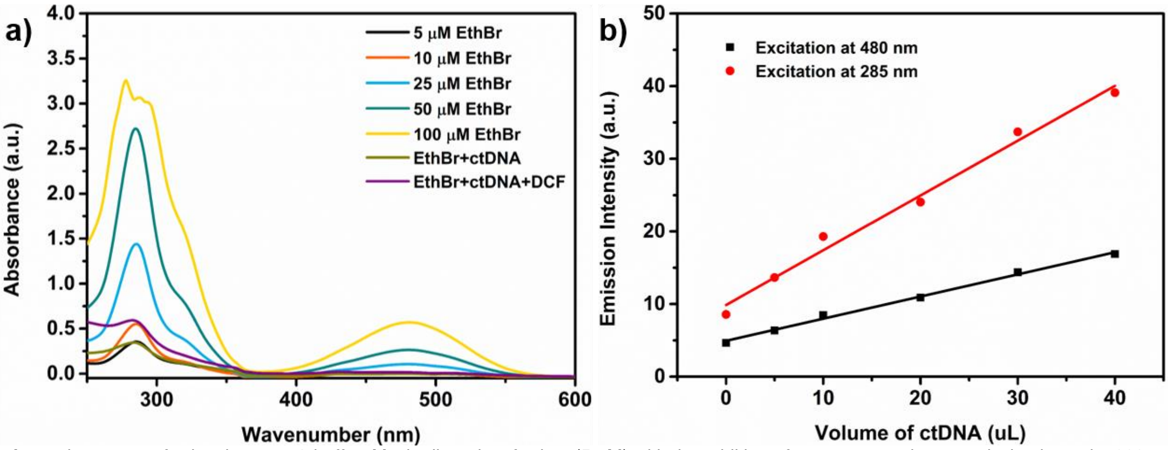

Figure S3. a) UV-vis spectra of EthBr in HEPES buffer; **b)** The linearity of EthBr (5 μM) with the addition of ctDNA at maximum emission intensity 620 nm, excited at 480 nm and 285 nm respectively.[1]

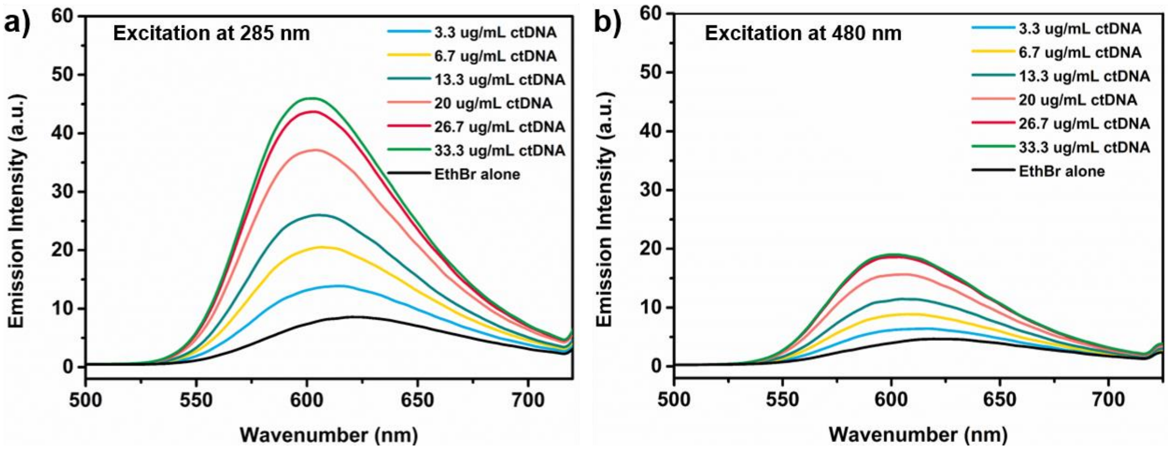

Figure S4. Fluorescence emission intensity of EthBr (5 μM) with the addition of ctDNA, excited at **a)** 285 nm and **b)** 480 nm respectively.

The binding behavior of DCFs with calf thymus DNA (ctDNA) and salmon sperm DNA (spDNA) was assessed at λ_{ex} 480 nm and λ_{em} 620 nm.

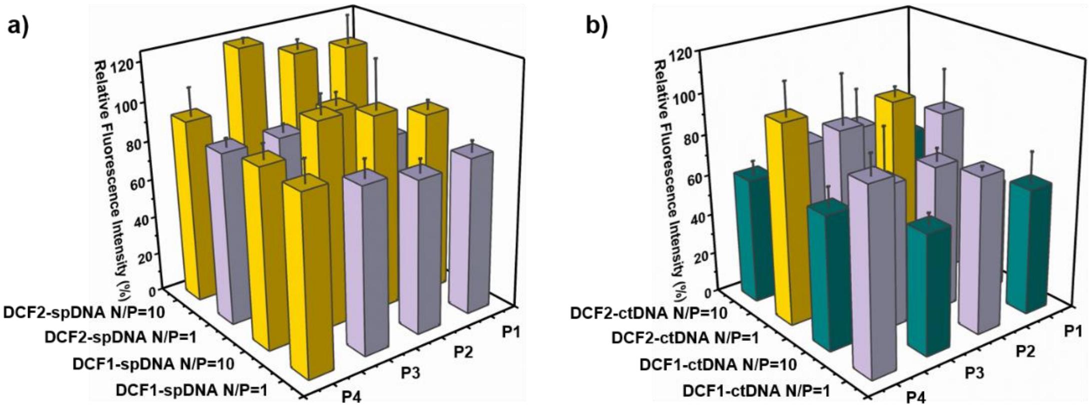

Figure S5. Relative fluorescence intensity of **a) DCFs-bPEI800-spDNA** complexes and **b) DCFs-bPEI800-ctDNA** complexes at fixed **N/P = 10, 1** with varied PEG chains: green 0-70%; purple 70-90%; yellow ＞90%.

3. Isothermal titration calorimetry

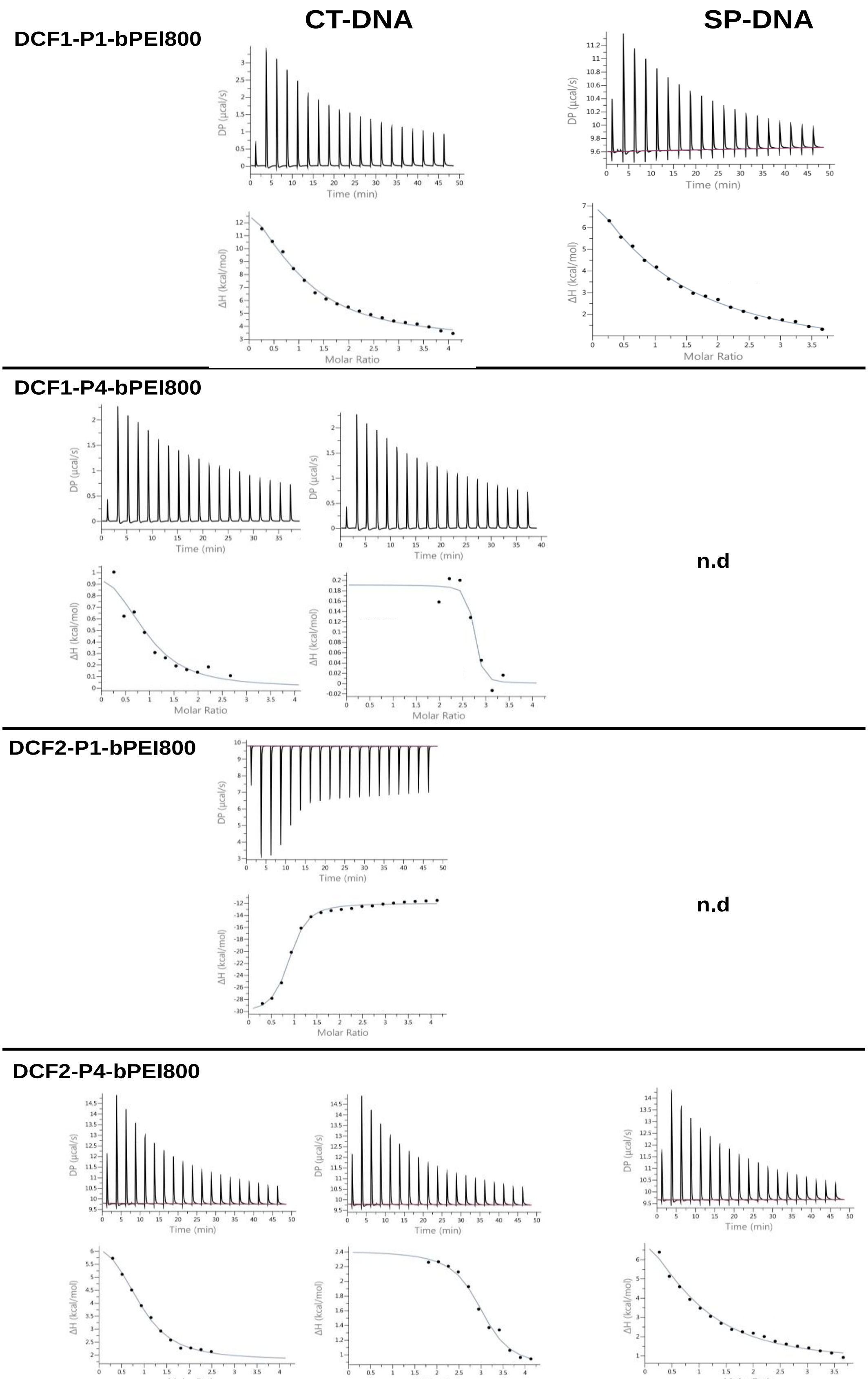

Figure S6. ITC binding isotherms: DNA binding raw data and curves after integration of individual heat flow signals as function of ligand/DNA molar ratio in the calorimeter cell.

4. Synthesis of Arginine building blocks (A1, A2, A3)

Arginine building blocks were synthesized by using a modifed resin and a previously-reported procedure of solid-phase peptide synthesis (SPPS)[2-3]:

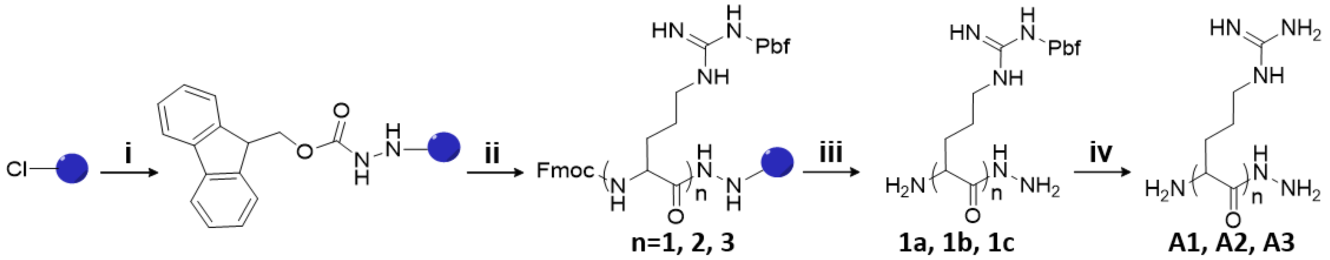

Scheme S1. General synthetic route of Arginine building blocks (**A1**, **A2**, **A3**). i) Resin modification, ii) SPPS, iii) Mild cleavage, iv) Deprotection.
Pbf: 2,2,4,6,7-pentamethyldlhydrobenzofuran-5-sulfonyl.

i. Preparation of the modified 2-chlorotrityl resin: To a stirred suspension of 2-chlorotrityl resin (10.5 g, 1.6 mmol Cl/g) in NMP/DMSO was added DIEA (14.7 mL, 84.3 mmol). After the adding of a solution of 9-fluorenylmethyl carbazate (12.84 g, 50.58 mmol), the mixture was stirred for 48 h at room temperature. The reaction mixture was filtered and the capping was finished by MeOH. Then the resin was washed with DMSO (3×), CH_2Cl_2 (2×), isopropanol (1×), CH_2Cl_2 (2×) and Et_2O (3×) and dried in vacuo.

ii. Peptide coupling procedure: The modified resin (0.62 g, 0.595 mmol/g) was suspended in CH_2Cl_2 for 15 min, then filtered and washed with DMF (3×). The Fmoc deprotection was performed by suspending the resin in piperidine/DMF (2/8) for 5 min, twice. After that, the resin was filtered, washed with DMF and dried in vacuo. Coupling was carried out by suspending the resin in a solution of Fmoc-Arg(Pbf)-OH (3 mL, 0.6 M in DMF, 5 eq.), HATU (9.2 mL, 0.2 M in DMF, 5 eq.), and DIEA (0.63 mL, 10 eq.). After 1 h, the resin was filtered, washed with DMF and the coupling procedure was finished twice. The resin was washed with DMF, and the Fmoc deprotection was performed by suspending in piperidine/DMF (2/8), 5 min, twice. The resin was filtered, washed with DMF and dried in vacuo. The second coupling and the third coupling were repeated with the same procedure of the first coupling. The Fmoc deprotection was performed by suspending in piperidine/DMF (2/8), 5 min, twice.

iii. Mild cleavage: The product was then cleaved in mild condition with a TFA/DCM (1:99) solution for 3 minutes for four times. And then the filtrate was neutralized with MeOH/Pyridine (8:2) and concentrated in vacuo. The crude product (**1a-1c**) was obtained after HPLC purification. A gradient was followed: Solution A: Water (0.1% TFA); Solution B: Acetonitrile (0.1% TFA); Solution C: Acetonitrile; Flow: 40 min/mL; Time: 55 minutes; Gradient: 5 min, 0% to 20% solvent B; 45 min, 20% to 80% solvent B; 50 min, 80% to 100% solvent B; 55 min, 100% solvent B. ‘
1a: Yield: 59%, 232 μmol. LC/MS: t_R 2.682 min. MS m/z calcd for [$C_{19}H_{32}N_6O_4S$] 440.22, found $[M+H]^+$ 441.00.
1b: Yield: 0.6%, 1.8 mg, 2.1 μmol. LC/MS: t_R 3.475 min. MS m/z calcd for [$C_{38}H_{60}N_{10}O_8S_2$] 848.40, found $[M+H]^+$ 849.20.
1c: Yield: 0.8%, 3.8 mg, 3.0 μmol. LC/MS: t_R 2.779 min. MS m/z calcd for [$C_{57}H_{88}N_{14}O_{12}S_3$] 1256.59, found $[M+2H+Na]^{3+}$ 427.00.

iv. 1a-1c were then deprotected with a TFA/TIS/H_2O (95:2.5:2.5) solution for 12 hours. The solution was concentrated, precipitated by adding Et_2O, and centrifuged. The supernatant was removed and the crude material was freeze-dried to give a white solid **A1**, **A2** and **A3**. The exact concentration was determined by ^1H NMR (D_2O) using tert-butanol as internal reference.
A1: Yield: 49%, 181 μmol. LC/MS: t_R 0.376 min. MS m/z calcd for [$C_6H_{16}N_6O$] 188.14, found $[M+H]^+$ 189.00.
A2: Yield: 0.15%, 0.56 μmol. LC/MS: t_R 0.409 min. MS m/z calcd for [$C_{12}H_{28}N_{10}O_2$] 344.24, found $[M+H]^+$ 345.05.
A3: Yield: 0.35%, 1.3 μmol. LC/MS: t_R 0.406 min. MS m/z calcd for [$C_{18}H_{40}N_{14}O_3$] 500.34, found $[M+2H+Na]^{3+}$ 175.10.

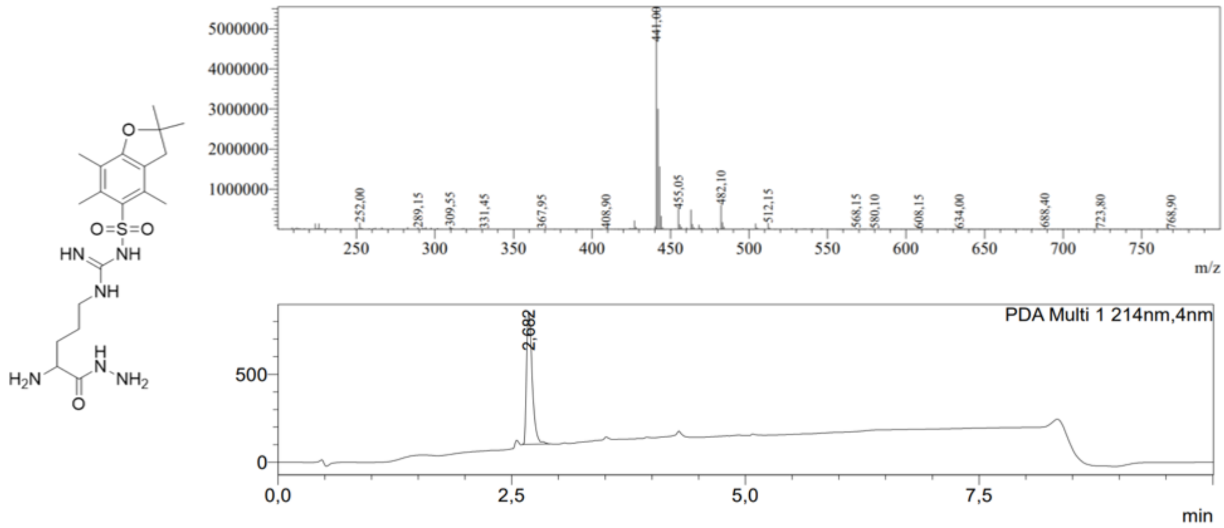

Figure S7. LC/MS analysis of **1a**: MS spectrum (top) and UV chromatogram (bottom).

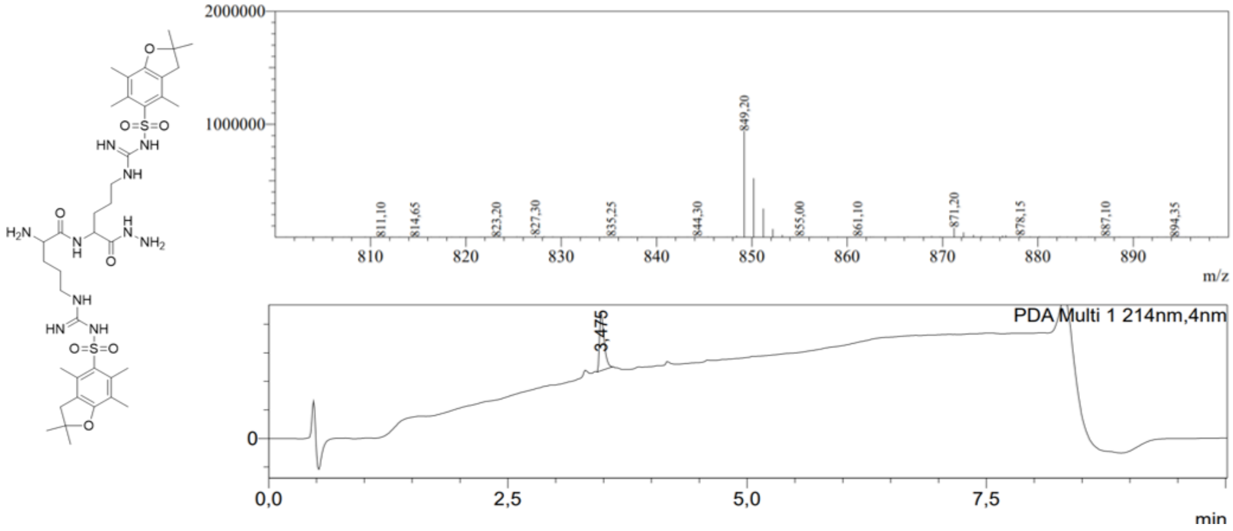

Figure S8. LC/MS analysis of **1b**: MS spectrum (top) and UV chromatogram (bottom).

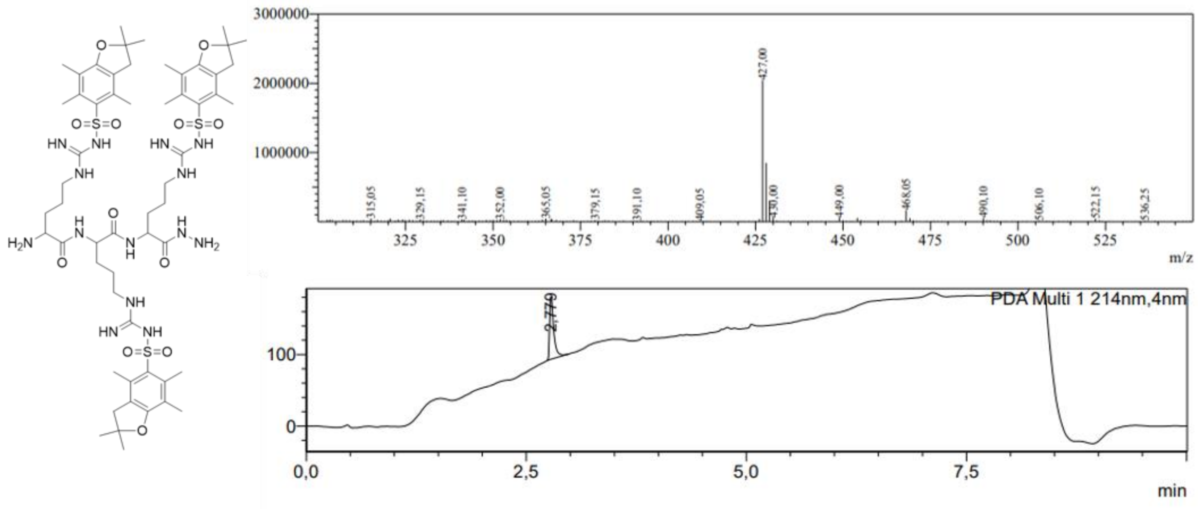

Figure S9. LC/MS analysis of **1c**: MS spectrum (top) and UV chromatogram (bottom).

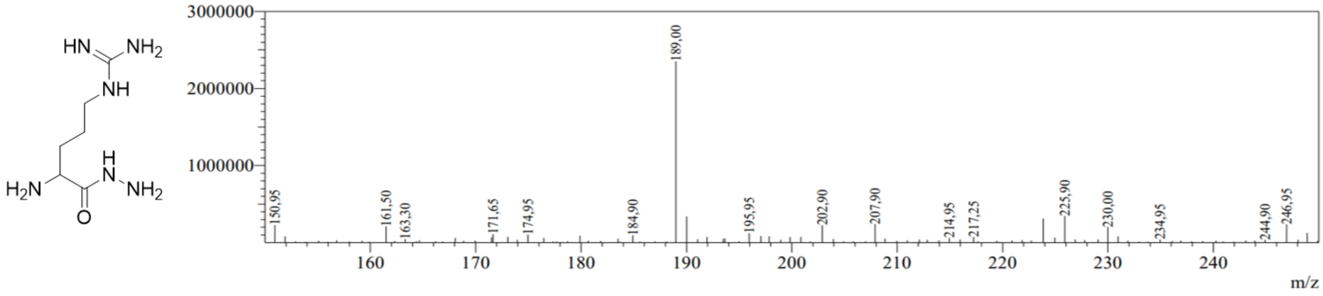

Figure S10. LC/MS analysis of **A1**: MS spectrum (top) and UV chromatogram (bottom).

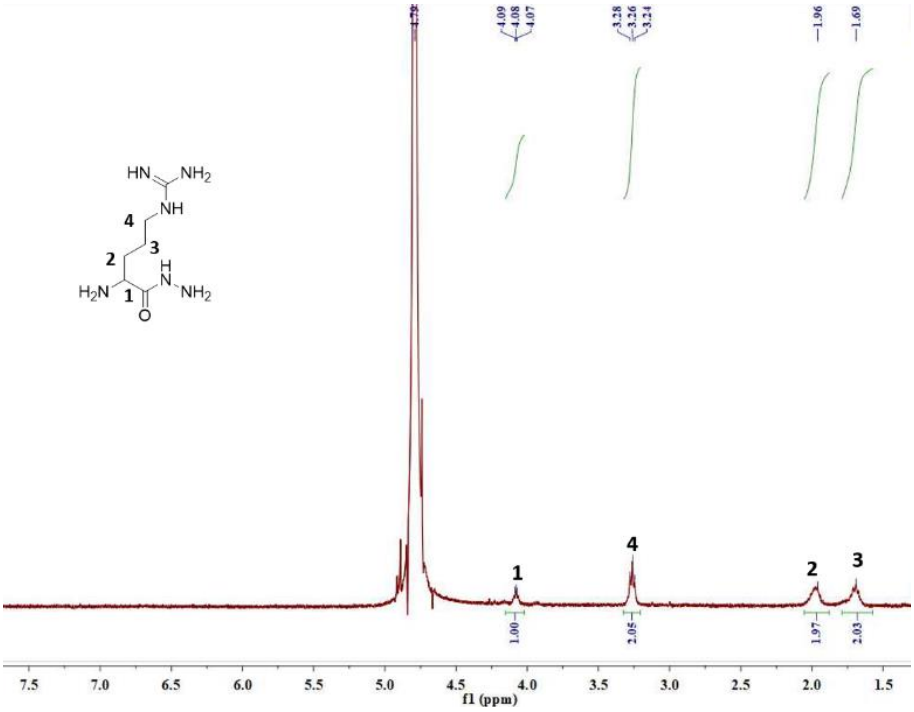

Figure S11. ^{1}H NMR spectrum of **A1** in D_2O.

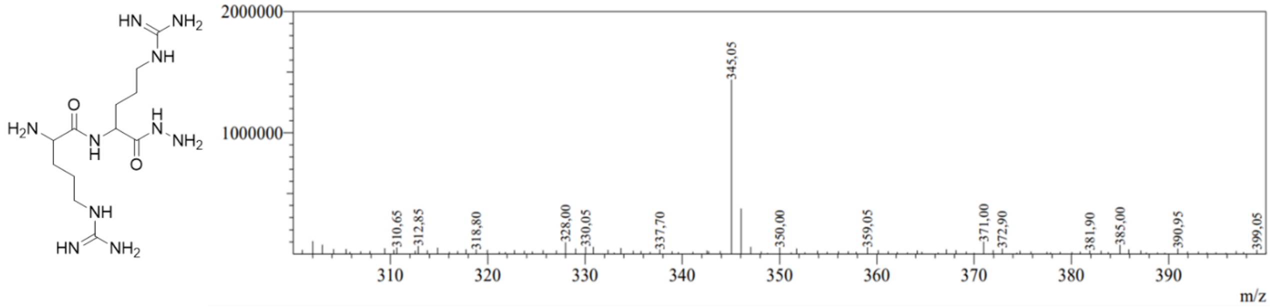

Figure S12. LC/MS analysis of **A2**: MS spectrum (top) and UV chromatogram (bottom).

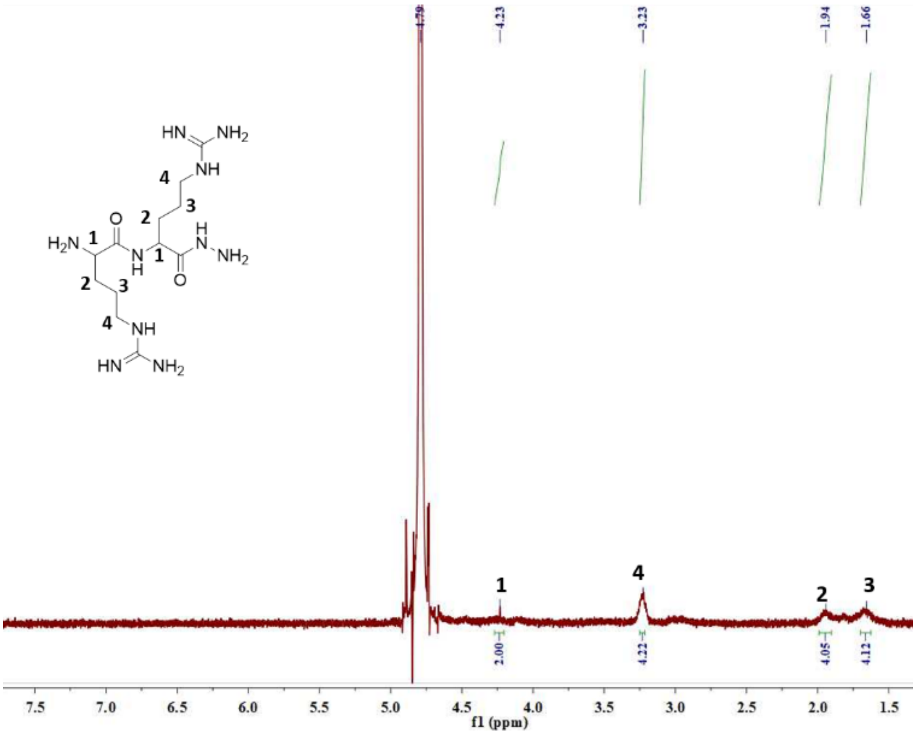

Figure S13. ^{1}H NMR spectrum of **A2** in D_2O.

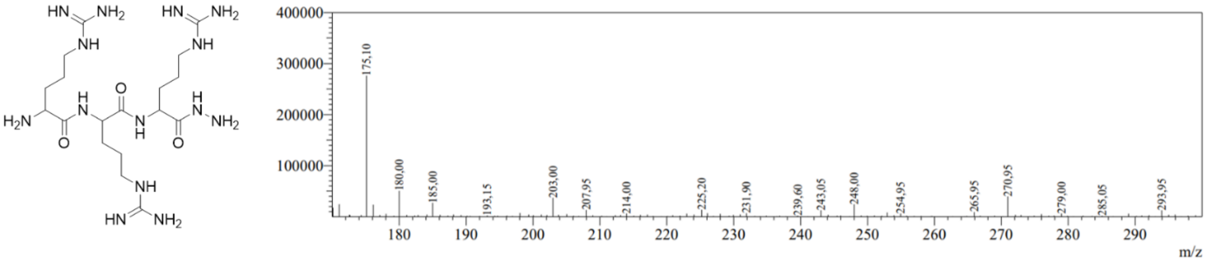

Figure S14. LC/MS analysis of **A3**: MS spectrum (top) and UV chromatogram (bottom).

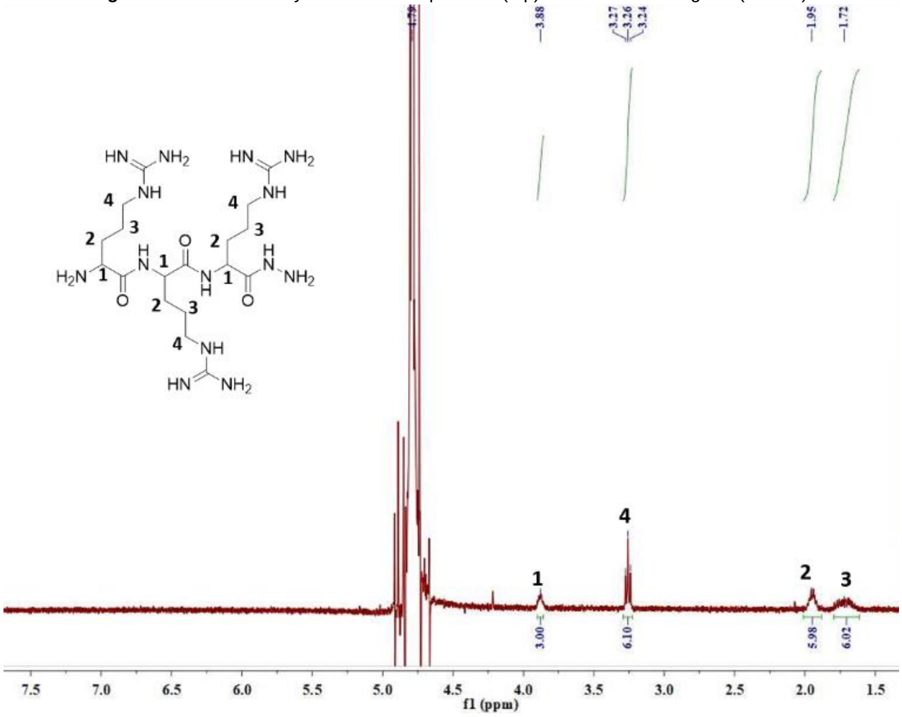

Figure S15. ^{1}H NMR spectrum of **A3** in D_2O.

5. Preparation of dynamic constitutional frameworks

5.1 Synthesis of SQ-PEG

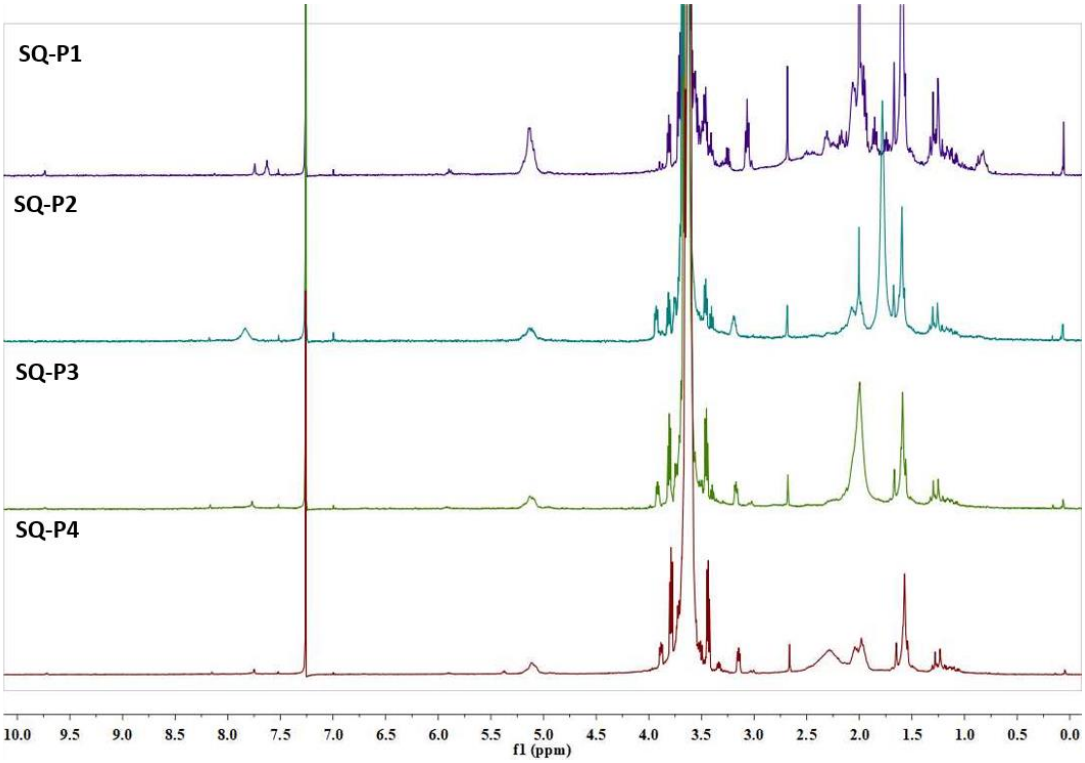

Figure S16. ^{1}H NMR spectrum of **SQ-PEG** in $CDCl_3$.

5.2 Synthesis of SQ-PEG-BTA

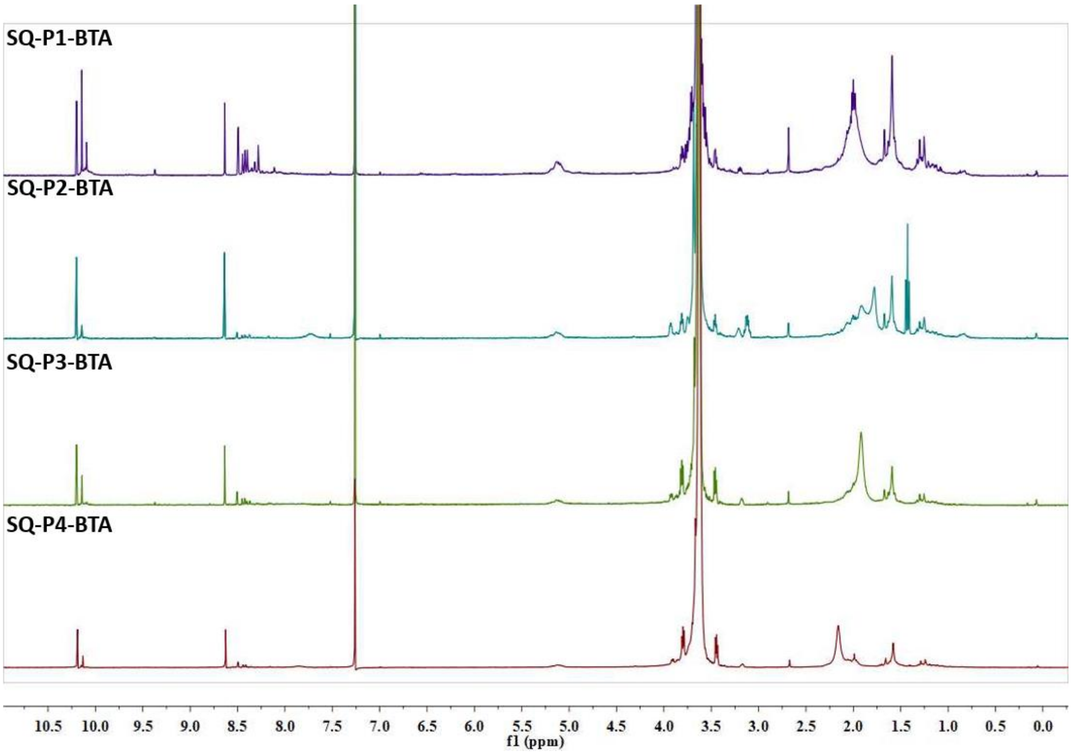

Figure S16. ^{1}H NMR spectrum of **SQ-PEG-BTA** in $CDCl_3$.

5.3 Preparation of DCF1

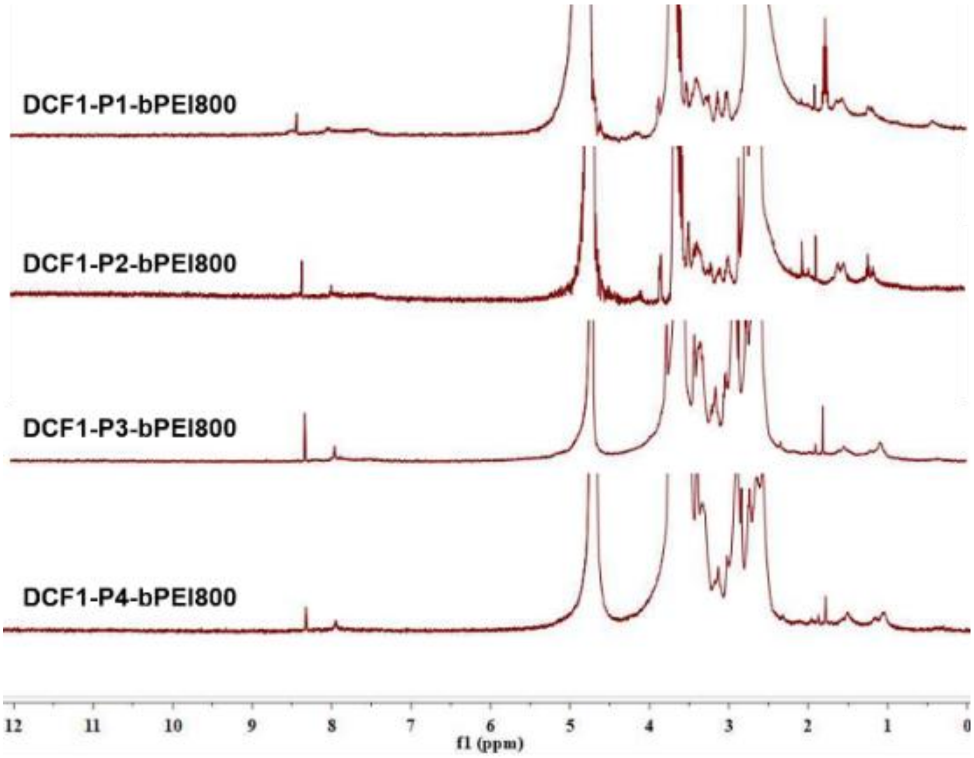

Figure S18. ^{1}H NMR spectrum of **DCF1-bPEI800** in D_2O.

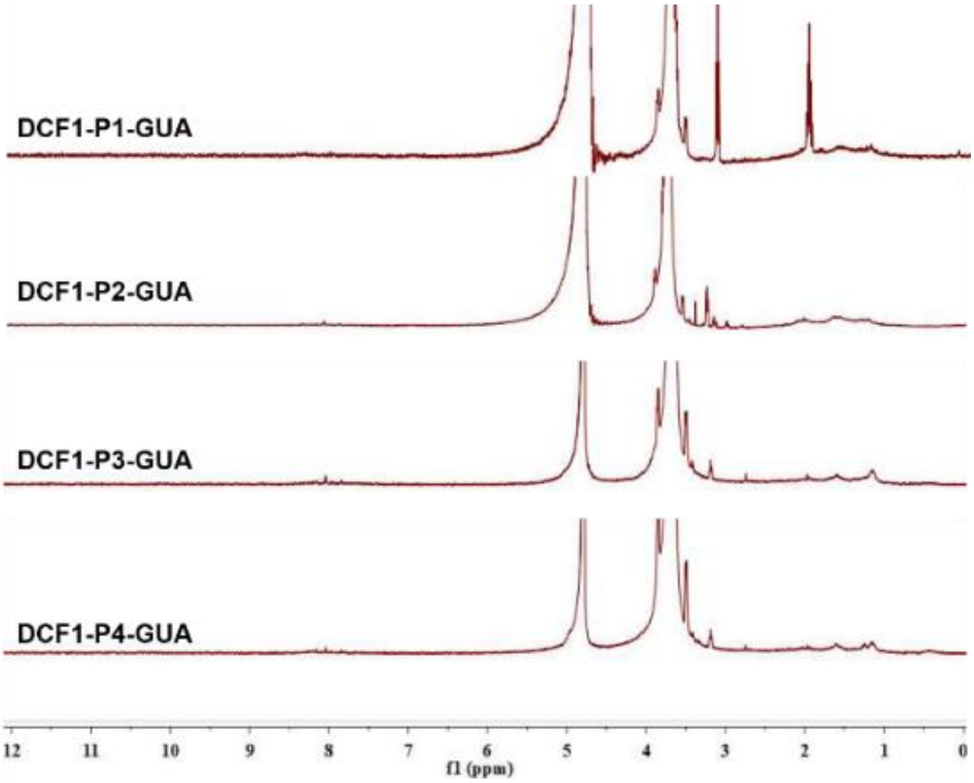

Figure S19. ^{1}H NMR spectrum of **DCF1-GUA** in D_2O.

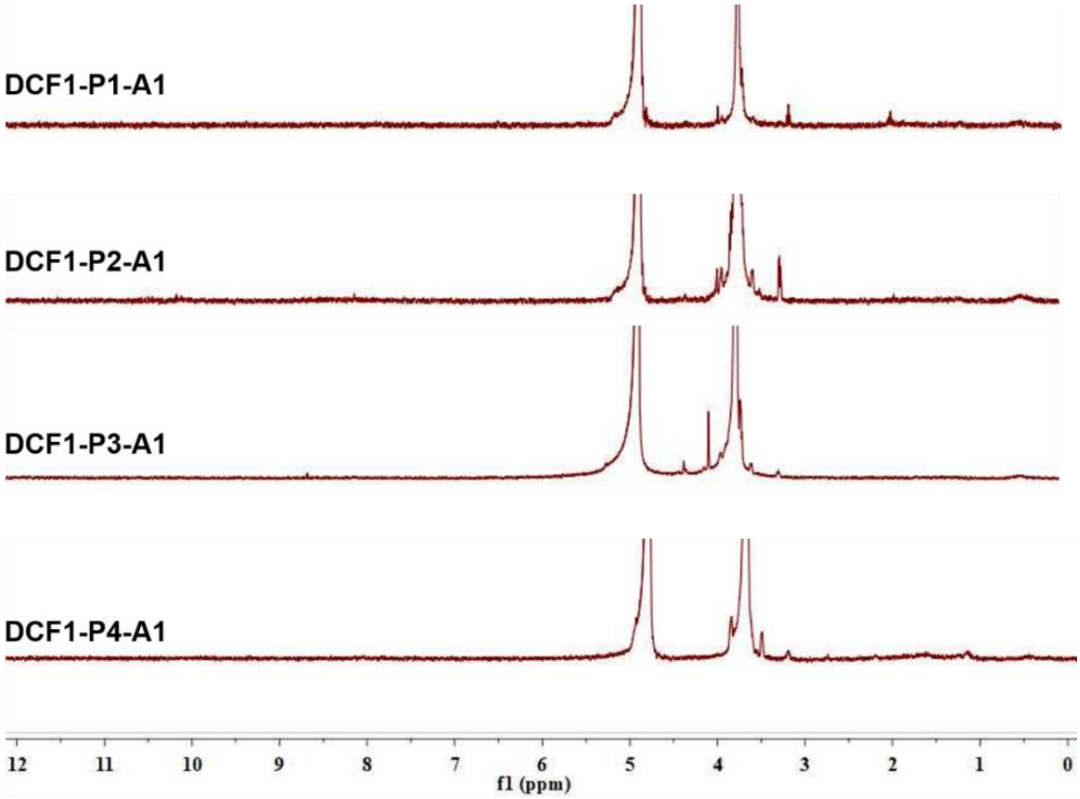

Figure S20. ^{1}H NMR spectrum of **DCF1-A1** in D_2O.

5.4 Synthesis of BTA-PEG

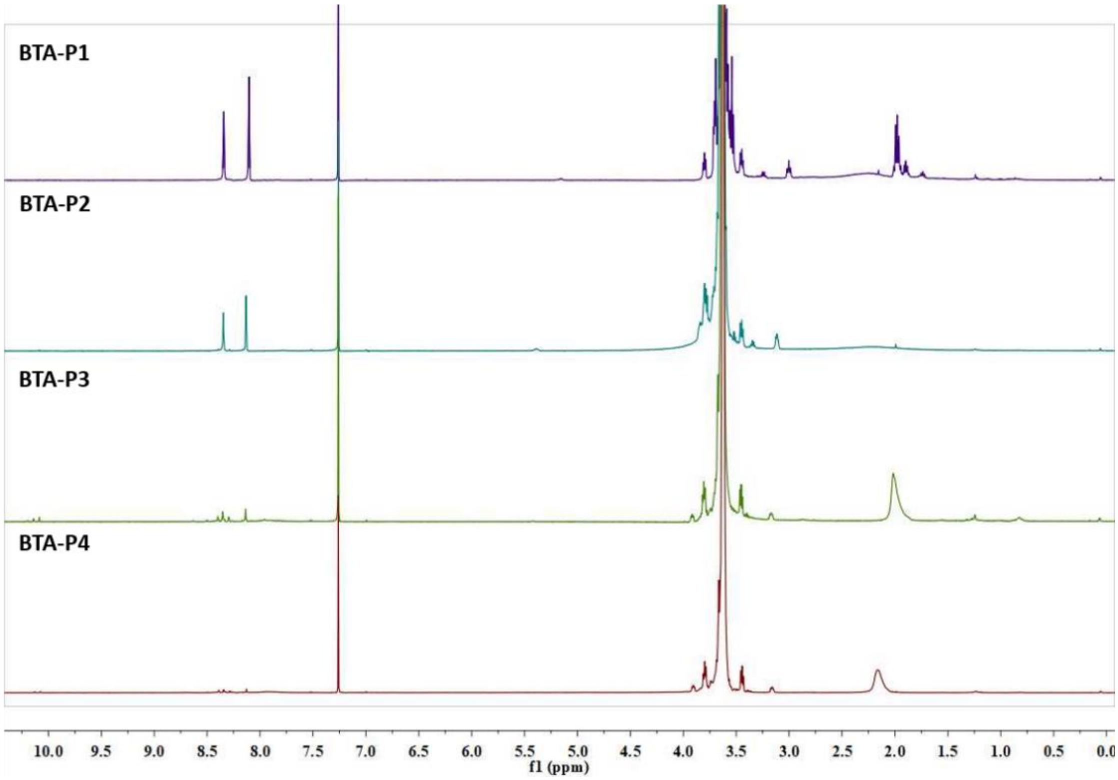

Figure S21. ^{1}H NMR spectrum of **BTA-PEG** in $CDCl_3$.

5.5 Synthesis of BTA-PEG-SQ

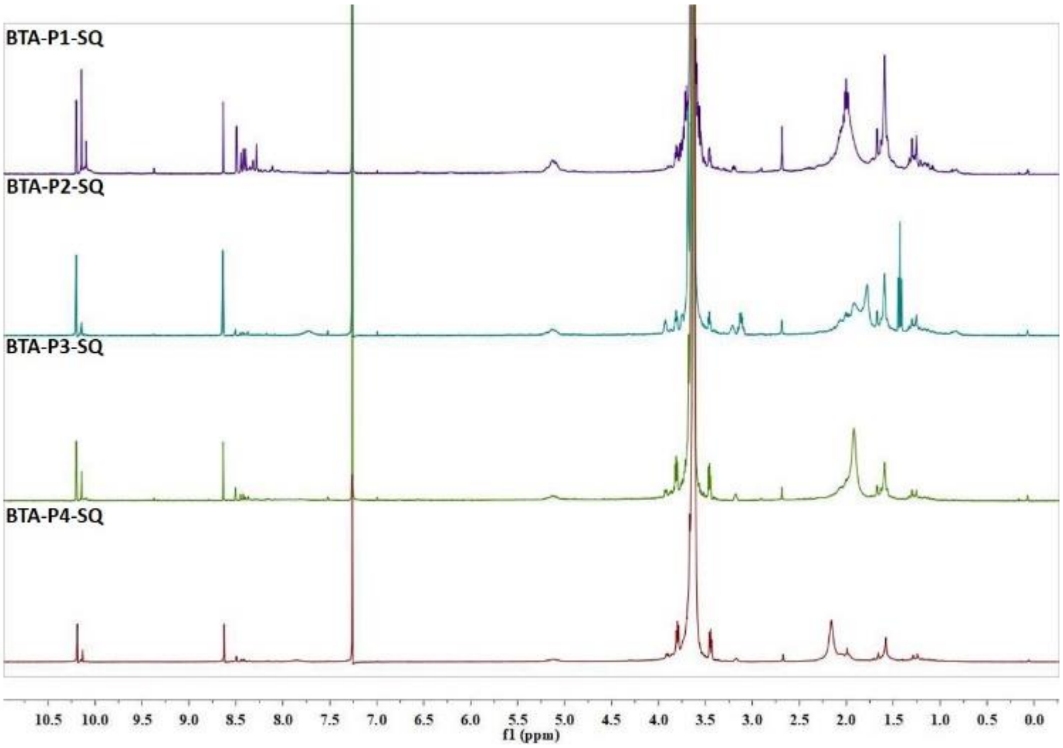

Figure S22. ^{1}H NMR spectrum of **BTA-PEG-SQ** in $CDCl_3$.

5.6 Synthesis of (BTA)3-(PEG)3-SQ

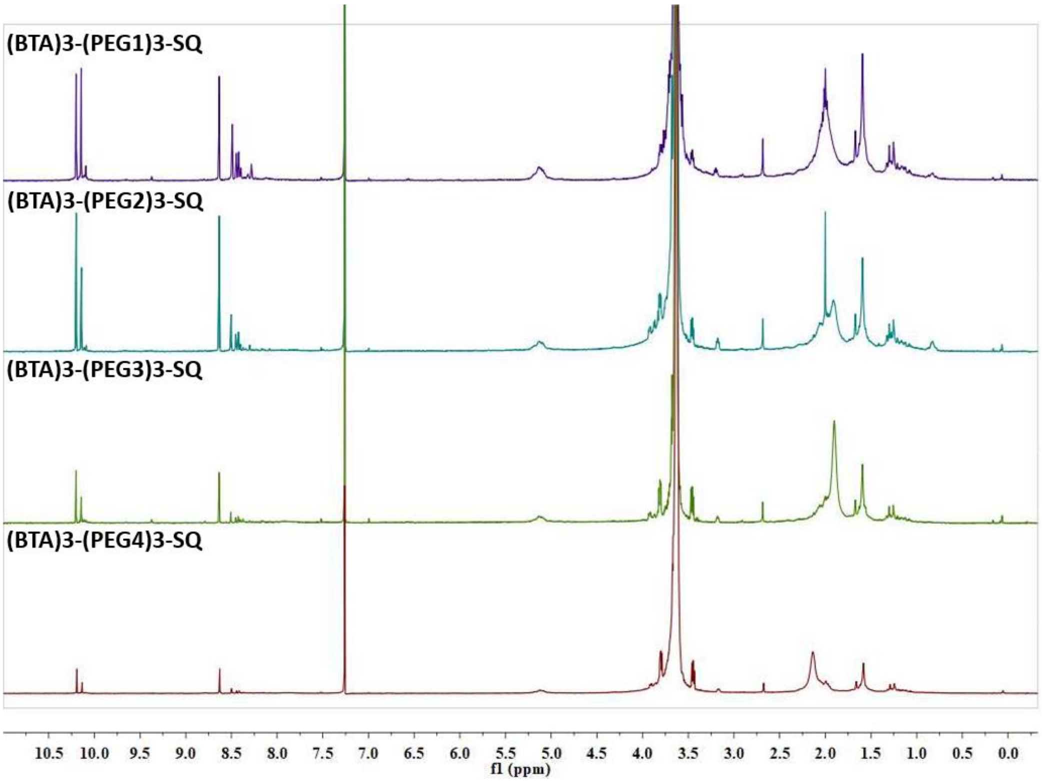

Figure S23. ^{1}H NMR spectrum of **(BTA)3-(PEG)3-SQ** in $CDCl_3$.

5.7 Preparation of DCF2

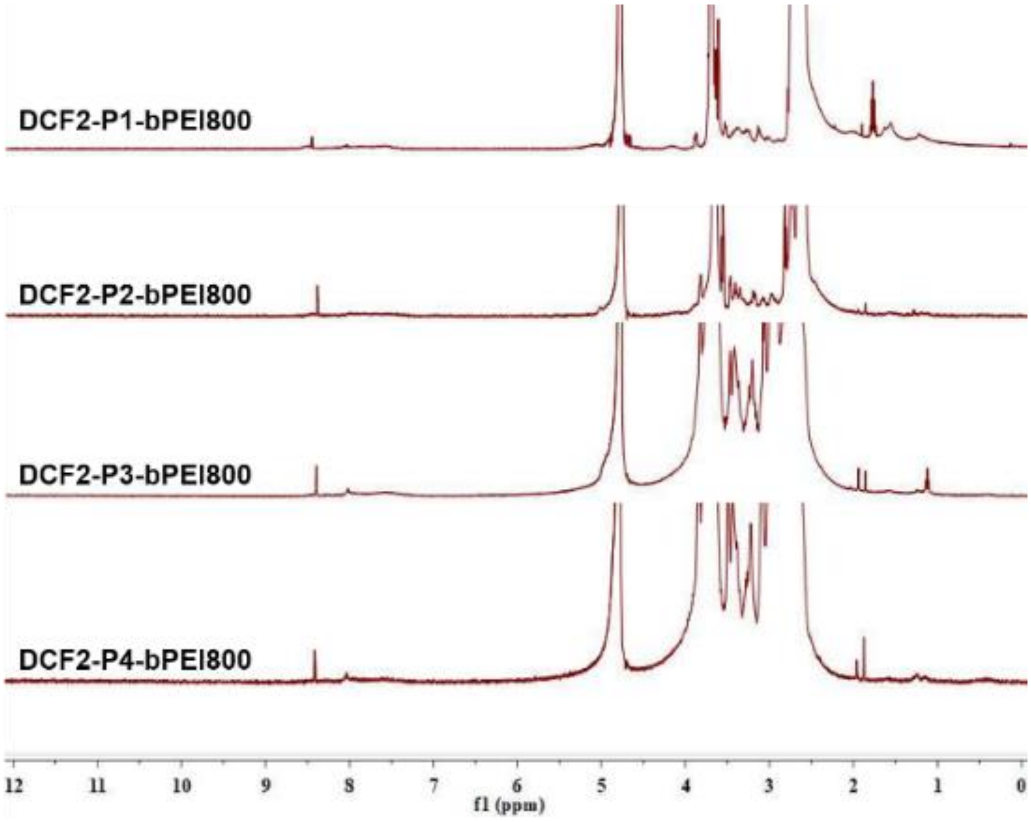

Figure S24. ^{1}H NMR spectrum of **DCF2-bPEI800** in D_2O.

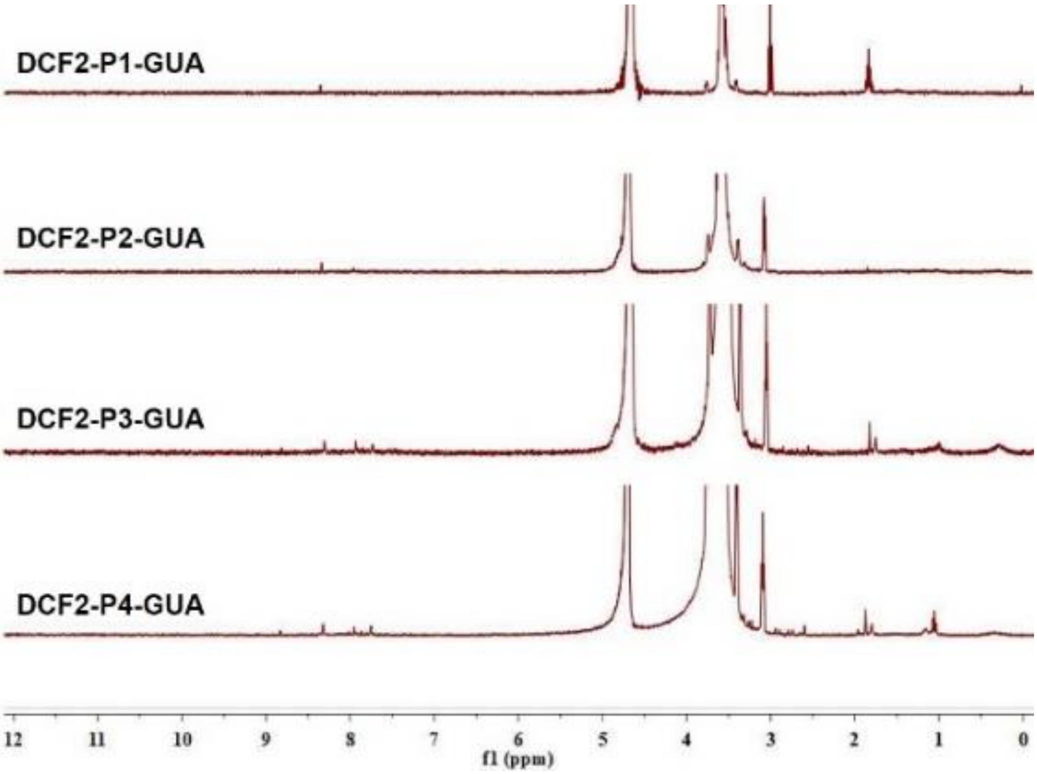

Figure S25. ^{1}H NMR spectrum of **DCF2-GUA** in D_2O.

6. References

[1] J. Carbajo, C. Adán, A. Rey, A. Martínez-Arias, A. Bahamonde. *Appl. Catal. B: Environ.* **2011**, *102*, 85–93.

[2] E. Bartolami, Y. Bessin, N. Bettache, M. Gary-Bobo, M. Garcia, P. Dumy, S. Ulrich. *Org. Biomol. Chem.* **2015**, 13, 9427-9438.

[3] N. Laroui, M. Coste, D. Su, L. Ali, Y. Bessin, M. Barboiu, M. Gary-Bobo, N. Bettache, S. Ulrich. *Angew. Chem., Int. Ed.* **2020**, 11, 5783-5787.
